# Microbe-aided thermophilic composting accelerates manure fermentation

**DOI:** 10.3389/fmicb.2024.1472922

**Published:** 2024-10-25

**Authors:** Likun Wang, Yan Li, Xiaofang Li

**Affiliations:** ^1^Center for Agricultural Resources Research, Institute of Genetics and Developmental Biology, Chinese Academy of Sciences, Shijiazhuang, China; ^2^Yancheng Institute of Soil Ecology, Yancheng, China; ^3^College of Advanced Agricultural Sciences, University of Chinese Academy of Sciences, Beijing, China

**Keywords:** manure, thermophilic composting, microbe-aided, thermophiles, comparative genomics

## Abstract

Aerobic composting is a key strategy to the sustainable use of livestock manure, which is however constrained by the slow kinetics. Microbe-aided thermophilic composting provides an attractive solution to this problem. In this study, we identified key thermophilic bacteria capable of accelerating manure composting based on the deciphering of manure bacterial community evolution in a thermophilic system. High-throughput sequencing showed a significant evolution of manure bacterial community structure with the increasing heating temperature. Firmicutes were substantially enriched by the heating, particularly some known thermotolerant bacterial species, such as *Novibacillus thermophiles*, *Bacillus thermolactis*, and *Ammoniibacillus agariperforans*. Correspondingly, through function prediction, we found bacterial taxa with cellulolytic and xylanolytic activities were significantly higher in the thermophilic process relative to the initial stage. Subsequently, a total of 47 bacteria were isolated *in situ* and their phylogenetic affiliation and degradation capacity were determined. Three isolates were back inoculated to the manure, resulting in shortened composting process from 5 to 3 days with Germination Index increased up to 134%, and improved compost quality particularly in wheat growth promoting. Comparing to the mesophilic and thermophilic *Bacillus*, the genomes of the three isolates manifested some features similar to the thermophiles, including smaller genome size and mutation of specific genes that enhance heat tolerance. This study provide robust evidence that microbe-aided thermophilic composting is capable to accelerate manure composting and improve the quality of compost, which represents a new hope to the sustainable use of manure from the meat industry.

## Introduction

Waste recycling and reuse to achieve carbon neutrality is a goal that many countries are striving for (Burnley et al., 2011). Among the most common wastes, livestock manure from the growing livestock and poultry breeding industries becomes one of the primary contributors to non-point source environmental contamination (Sun et al., 2012). Correct treatment of livestock manure is thus crucial to addressing these challenges.

Landfill, incineration, anaerobic digestion, and aerobic composting are widely implemented technologies for organic solid waste treatment (Wainaina et al., 2020; De la Cruz et al., 2021; Vlaskin and Vladimirov, 2018), among which aerobic composting is currently one of the most widely used approaches for treating livestock and poultry manure (Pajura, 2023). Aerobic composting is a self-heating, dynamic, and complex biochemical process, during which the successful biotransformation of organic substrates is completed by many different microorganisms. Aerobic composting is considered as an effective technique for waste management and production of organic fertilizers that can significantly increase soil fitness (Chaurasia et al., 2018). However, a mature compost product that produced by traditional composting is usually completed in 90–270 days (Khalil et al., 2008; Zhang et al., 2013). Besides, numerous greenhouse gasses (GHG) were usually released during this process, including carbon dioxide (CO_2_) from organic carbon digestion and methane (CH_4_) from poor air circulation and low moisture inside the pile (Lin et al., 2022).

Different from the conventional composting, thermophilic composting provides the benefit of improving biotransformation efficiency and suppressing the proliferation of pathogenic microbes by continuously sustaining high substrate temperatures through external heating. For instance, Zaman et al. (2022) developed a procedure of thermophilic composting with auxiliary heating to 55°C, resulting in a significantly rapid composting process and lower CH_4_ emission (Zaman et al., 2022). Aerobic composting bio-reactors can be used to quickly heat up and maintain a high temperature state, which leads to shortened fermentation period to within 10 days, high efficiency, regulated reaction conditions, and centralized exhaust gas treatment.

In most enhancement approaches for livestock manure treatment, biological methods, either the addition of co-substrate or microbial inoculation technology, are often considered as the less expensive and environmental-friendly option (Dong et al., 2023; Niu et al., 2022). A growing body of experimental and observational literature is providing evidence that microbial inoculum has enhanced the composting process and improved maturity of waste and manure (Liu et al., 2023a). Microbial inoculation technique enhances the composting process by increasing the microbial population or ensuring that the appropriate microbial population is present to provide the required enzyme for digesting organic compounds. Cellulases and xylanases generated by microbes in compost catalyze the breakdown of about half of the available xylan and cellulose during the thermophilic phases of composting (Jurak et al., 2015). The addition of cellulose, starch and protein-degraded bacteria in swine manure and rice straw was reported to hasten composting by enhancing compost maturation (Wang and Liang, 2021). On the other hand, a number of studies have found that microbial inoculant has little to no substantial effect on compost maturity, due primary to the incompatibility of the exogenous microbial inoculums with the characteristics of the feedstock and the less-than-ideal operating conditions for composting. Thus, use of *in situ* isolated functional microbes will better adapt to the target environment (Gu et al., 2024), can be one of the strategies to improve the application efficiency of aerobic composting. Ma et al. (2022) indicated that the coupling effect of high temperature and thermophilic bacteria expedited the decomposition of organic materials and promoted the humification process. However, the information on the microbial dynamics during the thermophilic composting process are still missing. Such information is essential to isolate extreme heat-tolerant microbial strains that can be applied in thermophilic composting process, reduce the energy consumption and to increase the production rate of compost within a short time.

In the current study, high-throughput amplicon sequencing analysis was used to obtain a deep understanding of bacterial population dynamics during thermophilic composting process of consortia with cattle dung and chicken manure. Heat-tolerant bacterial strains with cellulose and lignin degrading properties were isolated indigenously based on microbial community dynamics. These isolates were used as microbial additives to enhance the composting kinetics of livestock manure. Physiochemical features of the compost and the fertilizer effect of the compost on wheat growth were evaluated to derive the degree of decomposition at various stage and quality of the matured compost. These results were expected to help identify core microbial species in strengthen fermentation in a livestock manure thermophilic composting system and provide valuable insights for the industrial-scale application of fermentation promoting microbes.

## Materials and methods

### Thermophilic composting process and sampling

Raw organic materials used for composting consisted of chicken manure and cow dung at a rate of 1:1 collecting from local farm of Yancheng, China. The raw materials were mixed with straw at a rate of approximately 15% (weight/weight). The properties of the raw materials are given in Supplementary Table S1. After thorough mixing, 1 kg of raw materials were sealed in a 2 L Mason jar and incubated in electro heating standing temperature cultivator (DH3600II, Taisite, Tianjin, China) with eight replicate jars for each treatment. A rapid heating composting strategy was adopted in this study to simulate the internal conditions of bioreactor. The composting process lasted a total of 6 days, including 5 days of heating-up and thermophilic process and 1 day of cooling process. The temperature in the incubator was gradually elevated by 5°C per hour from the initial 25°C to the highest temperature 60°C. Raw materials were subsequently incubated under 60°C for 5 days before gradually cooling down. Samples were collected at the initial stage (0 d), heating-up stage (1 d), thermophilic stage (3 d), and cooling stage (6 d). Aliquote of the manure samples were stored immediately in −70°C for molecular characterizations.

### Metagenomic sequencing and microbial community analysis

Eight compost samples from each of the four composting steps were collected and sent to Shanghai Majorbio Bio-pharm Technology Co., Ltd. for amplicon sequencing (Yan et al., 2024). Following the manufacturer’s instructions, the E.Z.N.A.^®^ soil DNA Kit (Omega Bio-tek, Norcross, GA, United States) was used to extract microbial community genomic DNA from composting samples. The hypervariable region V3-V4 of the bacterial 16S rRNA gene were amplified with primer pairs 338F (5’-ACTCCTACGGGAGGCAGCAG-3′) and 806R (5’-GGACTACHVGGGTWTCTAAT-3′). Purified amplicons were pooled in equimolar and performed paired-end sequencing on an Illumina MiSeq PE300 platform (Illumina, San Diego, United States) in accordance to the standard protocols by Majorbio. The raw reads were deposited into the NCBI Sequence Read Archive (SRA) database (Accession number: SRP513095).

The raw 16S rRNA gene sequencing reads were demultiplexed, quality-filtered by fastp version 0.20.0 (Chen et al., 2018) and merged by FLASH version 1.2.7 (Magoc and Salzberg, 2011). The direction of the sequences were adjusted and barcodes were extracted. Operational taxonomic units (OTUs) with 97% similarity cutoff (Edgar, 2013) were clustered using UPARSE version 7.1 (Edgar, 2013), and chimeric sequences were identified and removed. The taxonomy of each OTU representative sequence was analyzed by RDP Classifier version 2.2 (Wang et al., 2007) against the 16S rRNA database Silva v138 with a confidence threshold of 0.7.

The Majorbio online analysis platform was used to calculate the alpha diversity indices (Richness, Shannon, Chao1, Simpson index) and beta diversity metrics based on the shared presence (Jaccard distance) or abundance (Bray-Curtis distance) of taxa. These metrics were then ordinated via Principal Coordinates Analysis (PCoA). Direct gradient analysis, distance-based redundancy analysis, and permutative ANOVA were used to test for separation (10,000 permutations). The taxonomy differential analysis was conducted using non parametric Kruskal Wallis sum-rank test among each two pairs of the four steps. Random forest analysis was used to identify the key OTUs that can distinguish differences between the two groups of composting steps, as well as their impact on the model. The network analysis of bacterial community was conducted though Random Matrix Theory (RMT)-based network construction methods (Deng et al., 2012).^1^ The prediction of community metagenomic functional abundance was performed using PICRUSt1 and FAPROTAX.

### Microbial isolation

Microbes were isolated from thermophilic and maturated stage samples. Three separate 1 g compost sub-samples were obtained from each sample and individually placed in tubes containing 10 mL sterile distilled water. Compost suspensions were prepared by mixing on a vortex mixer for 60s, serially diluted, and spread onto nutrition agar (Aoboxing Bio-tech Co., Ltd., Beijing, China) plates for isolating bacteria. Culturing plates were incubated at 45°C and 60°C for isolating heat tolerant and extreme heat tolerant microorganisms. Colonies that can grow at 45 or 60°C were transferred to new media and morphologically redundant ones were removed. The left colonies were subjected to DNA extraction and molecular identification based on 16S sequences. The 16S rDNA sequences of the bacterial isolates were deposited into NCBI GenBank database with the Accession Numbers of PQ462071 to PQ462113 and PQ459755-PQ459762.

### Enzymatic assessment

For cellulase assay, the bacterial isolates were incubated on sodium carboxymethyl cellulose medium at 45°C for 18 h. Congo red [0.1% (volume/volume); Macklin, Shanghai, China] staining was performed for 5 min, and then the destaining process was carried out by soaking the stained sample in 1.0 M NaCl for 10 min. Ligninase production activity was assessed by incubating bacterial isolates on nutrient agar medium containing aniline blue (Solarbio, Beijing, China), and the results were assessed 18 h post incubation. The enzyme activity and enzyme-degrading ability could be assessed by the size of the clear zone on the medium around the bacterial colonies (Teather and Wood, 1982).

### Microbial inoculation for thermophilic composting enhancement

Three bacterial inocula with targeted properties were selected for microbial-aided thermophilic composting experiment as previous described. The selected strains were incubated in liquid LB medium and shaken at 45°C, 150 r/min overnight. The inocula were collected by centrifuging, and fresh cells were inoculated individually in manure at a ratio of 1: 100 (weight/weight) with 3 replicates for each microbial treatment. A non-microbial inoculation control (NI) with 3 replicates was included in this experiment. Compost samples were collected at 3 days and 5 days post heat treatment.

### Analysis of compost’s physicochemical properties

Composting temperatures were recorded using a temperature sensor. Moisture was measured by drying fresh solid samples at 105°C for approximately 8 h to achieve a constant weight. Total carbon and total nitrogen were determined using an elemental analyzer (FlashSMART, Thermo Fisher Scientific Inc., MA, United States). Fresh solid samples were mixed with deionized water at a mass ratio of 1:10 and shaken for 1 h to obtain the water extract for the measurement of pH, electrical conductivity (EC), and seed Germination Index (GI). The pH and EC values were determined using a FiveEasy Plus™ pH/EC meter (Mettler Toledo, Shanghai, China). The GI was measured following the methods described previously by Wang et al. (2023) and Liu et al. (2023b). The GI values were measured using 10 cucumber seeds cultured in the water extract at 25°C for 48 h in darkness. Deionized water was used as a control. Organic matter content in the compost was determined by potassium dichromate volumetric method. In addition, total soluble phosphorus and total potassium contents were determined using UV-1900i spectrophotometers (Shimadzu Co., Ltd, Shanghai, China) and TAS-990 atomic absorption spectroscopy (Beijing Puxi General Instrument Co., Ltd, Beijing, China), respectively.

### Testing of manure quality by a wheat growth test

Composted manure was subjected to wheat growth tests to examine the quality of the fermentation products. After fermentation, composts from different treatments were thoroughly mixed with growing substrates at a volume ratio of 2:1. The growing substrates contained vermiculite and potting mix (Klasmann-Deilmann, Germany) at a ratio of 7:1 to create a barren nutritional environment. Wheat seeds were transplanted in the culture mix after germinating in Petri dish using distilled water. Five pots were applied for each compost sample with 2 seeds in each pot. Pots were arranged in a completely randomized design in an artificial climate growth chamber (PRX-800D-F, Ningbo, China). Plants were grown in the greenhouse at 25–28°C with a 14-h photoperiod, watered at 3-day intervals, and harvested 1 month after planting. Wheat seedlings shoot height, shoot, and root dry weight were measured at harvest.

### Bacterial whole genome sequencing

Genome sequencing was conducted at Shanghai Majorbio Bio-pharm Technology Co., Ltd. Genomic DNA was extracted using Bacterial DNA extraction kit (magnetic beads) (BioDynami, Alabama, United States) according to the manufacture’s protocol. Paired-end Illumina sequencing (2 × 150 bp) on Illumina Novaseq 6,000 (Illumina Inc., SanDiego, CA, United States) was used to sequence the whole-genome of FSB24, FSB30 and FSB35. The raw reads were deposited into the NCBI SRA database (Accession number: SRP513345).

The low-quality reads were filtered to obtain clean data using fastp 0.20.0 (Chen et al., 2018). After quality control, clean reads were *de novo* assembled to obtain genome draft using short sequence assembly software SOAP denovo2 (Luo et al., 2012), resulting in the optimal contigs assembly. Then, aligned contigs were locally assembled and optimized base on the paired-end and overlap relationships of reads to construct scaffold. Glimmer3 (Delcher et al., 2007), GeneMarkS-2 (Lomsadze et al., 2018), and Prodigal v2.6.3 (Hyatt et al., 2010) were used to predict the coding sequence (CDS) on the genome. tRNAscan-SE v2.0 (Chan and Lowe, 2019), Barrnap,^2^ and Tandem Repeats Finder v 4.09 (Benson, 1999) were used to predict tRNAs, rRNAs, and tandem repeat sequences, respectively. Genes were annotated against Gene Ontology (GO),^3^ Kyoto Encyclopedia of Genes and Genomes (KEGG),^4^ Cluster of Orthologous Groups of proteins (COG),^5^ KOG, evolutionary genealogy of genes: Non-supervised Orthologous Groups (eggNOG), Non-Redundant Protein Database (NR), Transporter Classification Database (TCDB), Swiss-Prot,^6^ Carbohydrate-Active enZYmes Database (CAZy) databases using diamond v2.1.9 (Buchfink et al., 2021) with an cutoff *E*-value of 1.0 e-5. Secondary metabolism gene cluster analysis was performed using antiSMASH v2.0.2 (Blin et al., 2013).

### Comparative genomics

Based on the genome maps of three isolates, their genes on genomes were compared to understand the functions, expression mechanisms, and evolution process. OrthoMCL v2.0 (Li et al., 2003) was used to obtain homologous gene families, as well as gene number and information in each gene family. The genomes of the three isolates FSB24, FSB30 and FSB35 were then screened for core genes (genes included in all isolates) and unique genes (genes included only in a specific isolate).

Genomic collinearity analysis and phylogenomic analysis were conducted by Mauve 2.4.0 (Darling et al., 2004). Isolates FSB24, FSB30 and FSB35 were aligned to 12 other *Bacillus* species, and also individually mapped to the known thermophile *Bacillus thermotolerans* (GCF_000812025.2) and mesophile *Bacillus cereus* (GCF_000007825.1) using BLAST+ with an E value ≤1e-5. Genes with identity <80% were considered as non-homolog genes. The phylogenetic relationship of the selected genes was constructed with MEGA 7.0 (Kumar et al., 2016) using the maximum likelihood method and 1,000 bootstrap replicates. Multiple sequence alignment was performed using ClustalW (Thompson et al., 1994), and p-distance was calculated.

### Statistical analysis

Plant growth and physicochemical compost properties data were subjected to ANOVA using SAS (Version 9.4; SAS Institute, Cary, NC) GLM model for a completely randomized design. Data were subjected to analysis of variance and means separation using Fisher’s least significant test, with *p* ≤ 0.05 considered significant.

## Results

### Succession in bacterial diversity during thermophilic composting process

**Figure 1:**
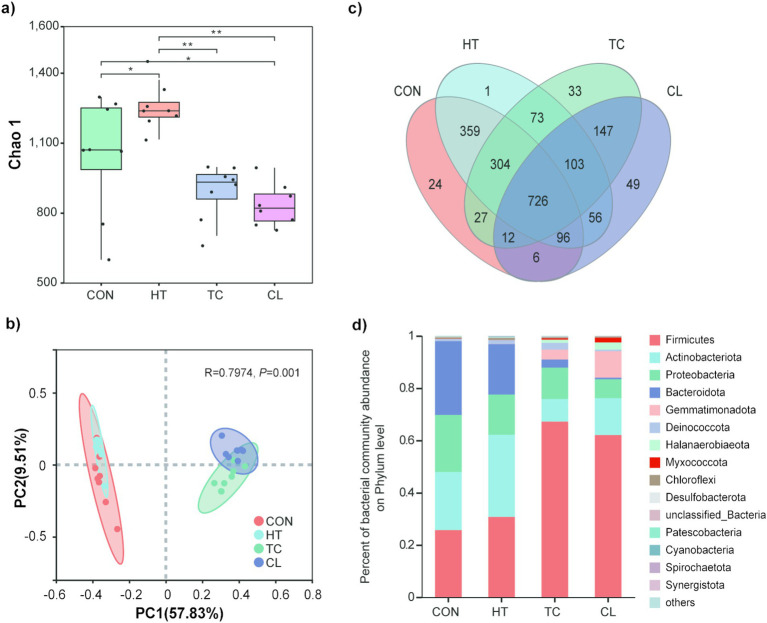
Abundance and diversity alteration of bacterial communities across the thermophilic composting process. **(a)** Chao 1 index of bacterial community across thermophilic composting processes. **(b)** Principal coordinates analysis of Bray-Curtis distances based on bacterial species abundance profiles across thermophilic composting. **(c)** Numbers of common and unique bacterial species across thermophilic composting showed by Venn diagram. **(d)** Relative abundance of bacteria in phylum level in the four steps of thermophilic composting.

The heat treatment resulted in decreasing Chao 1 indices of bacterial communities than the initial stage (Control). The Chao 1 indices of bacterial communities were not different between thermophilic (TC) and cooling (CL) steps, but they both significantly lower than heat-up (HT) step and the Control. In addition, the bacterial abundance, indicated by the Chao 1 index, in HT step was significantly higher than that in the Control (Figure 1a). PCoA analysis indicated that bacterial community compositions of the TC and CL steps were more similar to each other but distinct from that of the Control and HT (Figure 1b), additionally, the bacterial community compositions of Control and HT were more similar to each other. A total of 726 bacterial OTUs presented in all the composting steps, and individually, 24, 1, 33 and 49 OTUs were only presented in the Control, HT, TC and CL steps (Figure 1c). Among the 49 unique OTUs detected in CL process, OTU596 from the Genus of *Paenibacillus* (15.01%), OTU918 from the Genus of *Thermobacillus* (9.12%) and OTU556 from the Family of Limnochordaceae (8.71%) represented the greatest proportion among the unique OTUs. While among the unique OTUs in the Control, OTU63 from the Genus of *Brumimicrobium* (17.31%), OTU392 from the Family of Sphingobacteriaceae (9.62%), and the OTU47 from the Order of Peptostreptococcales-Tissierellales (9.62%) represented the greatest proportion.

Among bacterial phyla, Firmicutes, Actinobacteriota, Proteobateria, Bacteroidota, Gemmatimonadota, Deinococcota, Halanaerobiaeota, Myxococcota, Chloroflexi, and Desulfobacterota were detected at greater abundance in TC and CL processes (Figure 1d). The bacterial phylum that was significantly altered in abundance during the composting process was Firmicutes (Figure 1d), with about 2.5 fold increase in TC (67.21 ± 9.60%) and CL (62.5 ± 7.35%) steps relative to the Control (25.71 ± 14.27%). The abundance of Actinobacteriota was first increased and then decreased during the whole composting process. The abundances of Proteobacteria and Bacteroidota were decreased during composting, whereas the abundance of Gemmatimonadota was dramatically increased with the rising temperature (Figure 1d). Besides, the phyla Gemmatimonadota and Halanaerobiaeota were only in the top 10 abundant bacterial phyla of TC and CL, but not in HT or the Control.

### Differential analysis of bacterial taxonomy among the fermentation steps

Random forest analysis showed that the top 50 differentially abundant bacterial OTUs among the four fermentation steps were mainly in the phyla of Firmicutes, Proteobacteria, Bacteroidota, and Myxococcota (Supplementary Figure S2). Among them, the thermophilic bacteria *Ammoniibacillus agariperforans* (Sakai et al., 2015), *Novibacillus thermophiles* (Yang et al., 2015), and *Ureibacillus thermosphaericus* (Jia et al., 2017) were significantly abundant in the TC and CL steps. Whereas *Facklamia tabacinasalis* (Collins et al., 1999) and *Aerococcus urinaeequi* (Rasmussen, 2016), two potential human pathogens, were more abundant in HT step and the Control (Supplementary Figure S2).

**Figure 2:**
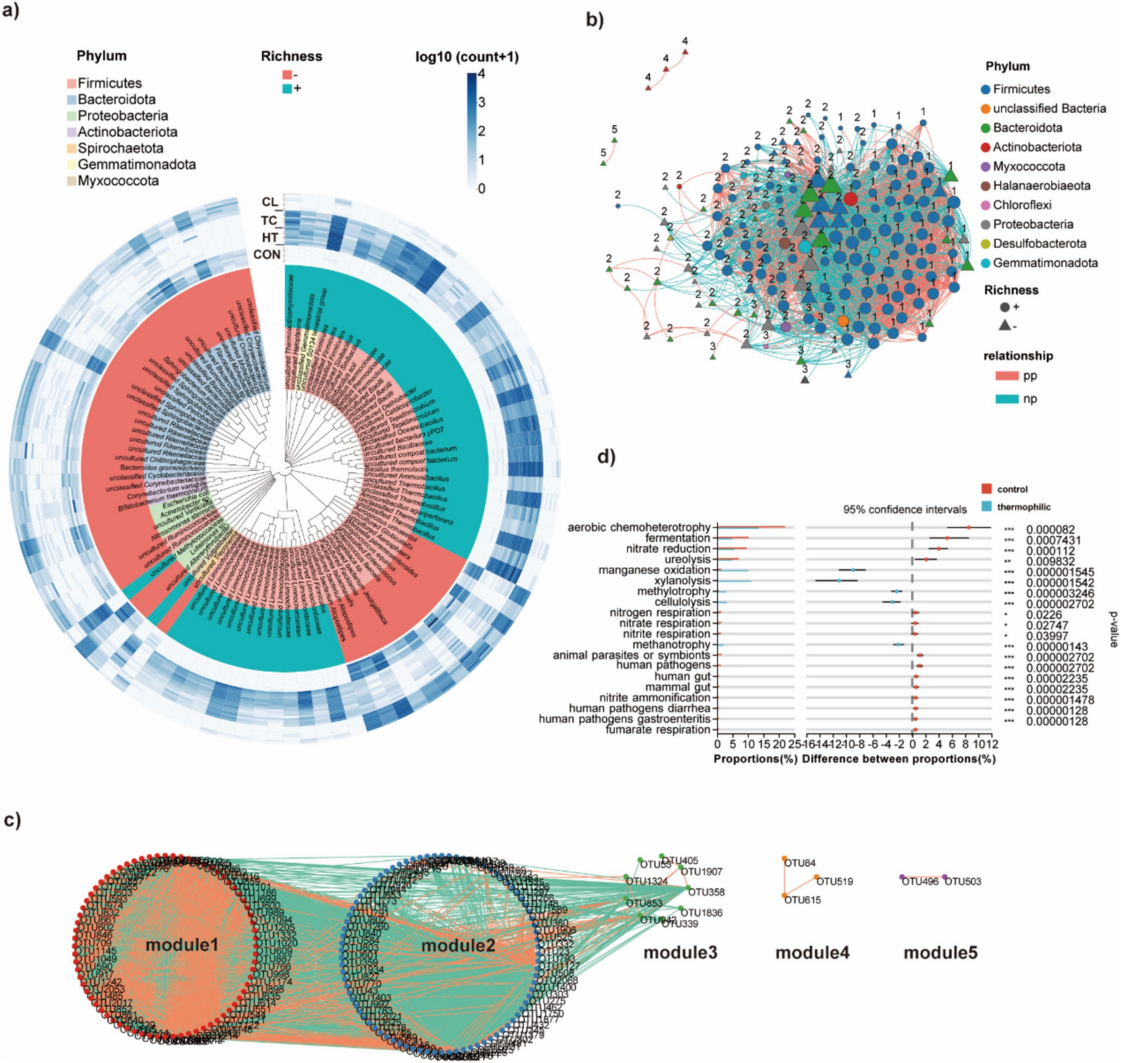
Pairwise comparison of bacterial taxonomy between the two groups (thermophilic group vs. control group). **(a)** The top 50 bacterial species that significantly altered in abundance comparing between thermophilic and control group. **(b)** Network of bacterial OTUs with significantly altered abundance, comparing between thermophilic and control group. **(c)** Modules of bacteria with significantly altered abundance, comparing between thermophilic and control group based on molecular ecological network (MENs) analysis. **(d)** Significantly different bacterial metabolites comparing between thermophilic and control group that analyzed through FAPROTAX.

Since bacterial communities in the TC and CL steps were similar to each other (so were HT and the Control) based on diversity and taxonomy analysis, samples from TC and CL (thermophilic group), as well as the Control and HT (control group) were combined to conduct the pairwise comparison. Through differential analysis of control group and thermophilic group, 464 differentially abundant bacterial OTUs were obtained. The top 50 significantly abundant bacteria in thermophilic group were mainly in the Phyla of Firmicutes, including the thermotolerant bacteria *Sinibacillus soli* (Yang and Zhou, 2014), *Bacillus thermolactis* (Coorevits et al., 2011), and *A*. *agariperforans* (Sakai et al., 2015) (Figure 2a). Besides, several bacteria in the Phyla of Gemmatimonadota, Myxococcota, and Proteobacteria were also significantly abundant in the thermophilic group (Figure 2a). The top 50 significantly abundant bacteria in the control group were mainly in the Phyla of Firmicutes, Bacteroidota, and Proteobacteria. Among them, *Streptococcus equinus* (Park et al., 2023), *F. tabacinasalis* (Collins et al., 1999), *A. urinaeequi* (Rasmussen, 2016), and *Enterococcus faecalis* (Kristich et al., 2014) from the Phylum of Firmicutes, and *Escherichia coli* from the Phylum of Proteobacteria are reported human/animal pathogens (Figure 2a).

Molecular ecological network (MENs) through RMT-based methods was constructed for all the differentially abundant bacterial OTUs between the thermophilic and control group, resulting in 172 nodes and 2,616 edges (Figure 2b). The 172 OTUs were classified into 5 modules, and OTU1379 (Firmicutes), OTU432 (Bacteroidota), and OTU491 (Bacteroidota) were in the centre of the network (Figure 2b). There were 72 bacterial OTUs classified to module 1 with 64 of them were significantly abundant in the thermophilic group, while eight of them were significantly abundant in the control group (Figure 2c). Among the 64 bacterial OTUs enriched in the thermophilic group, the slow growing species *Thermobifida fusca*, which was reported to promote the growth of other bacteria by sharing cobalamin in a quasi-natural composting system (Zhao et al., 2023), was related to 75 OTUs, and all the relationships were positive. There were 85 bacterial OTUs classified to module 2 with 44 of them were significantly abundant in the thermophilic group, while 41 of them were significantly abundant in the control group. The thermophilic species *Clostridiales bacterium* and *Bacillus thermocloacae* that significantly abundant in the thermophilic group had 1 negative and 3 positive connections with other OTUs, respectively. There were only three and two OTUs classified to module 4 and 5, respectively, and they were all significantly more abundant in the control group (Figure 2c).

FAPROTAX was used to map bacterial taxonomy to metabolic related functions. Through Wilcoxon rank-sum test, we found that the abundance of bacteria with metabolic functions of cellulolysis, xylanolysis, methanotrophy and manganese oxidation were significantly higher in the thermophilic group, while functions related to animal/plant pathogenesis were significantly higher in the control group (Figure 2d). Besides, functions related to nitrogen cycle, such as nitrite ammonification and nitrate reduction, were significantly decreased with temperature increasing (Figure 2d), suggesting the functions of bacterial community converted during the thermophilic composting process. It was consistent with previous reports (Song et al., 2016).

### High-throughput culturing of thermophilic fermentation strains

**Figure 3:**
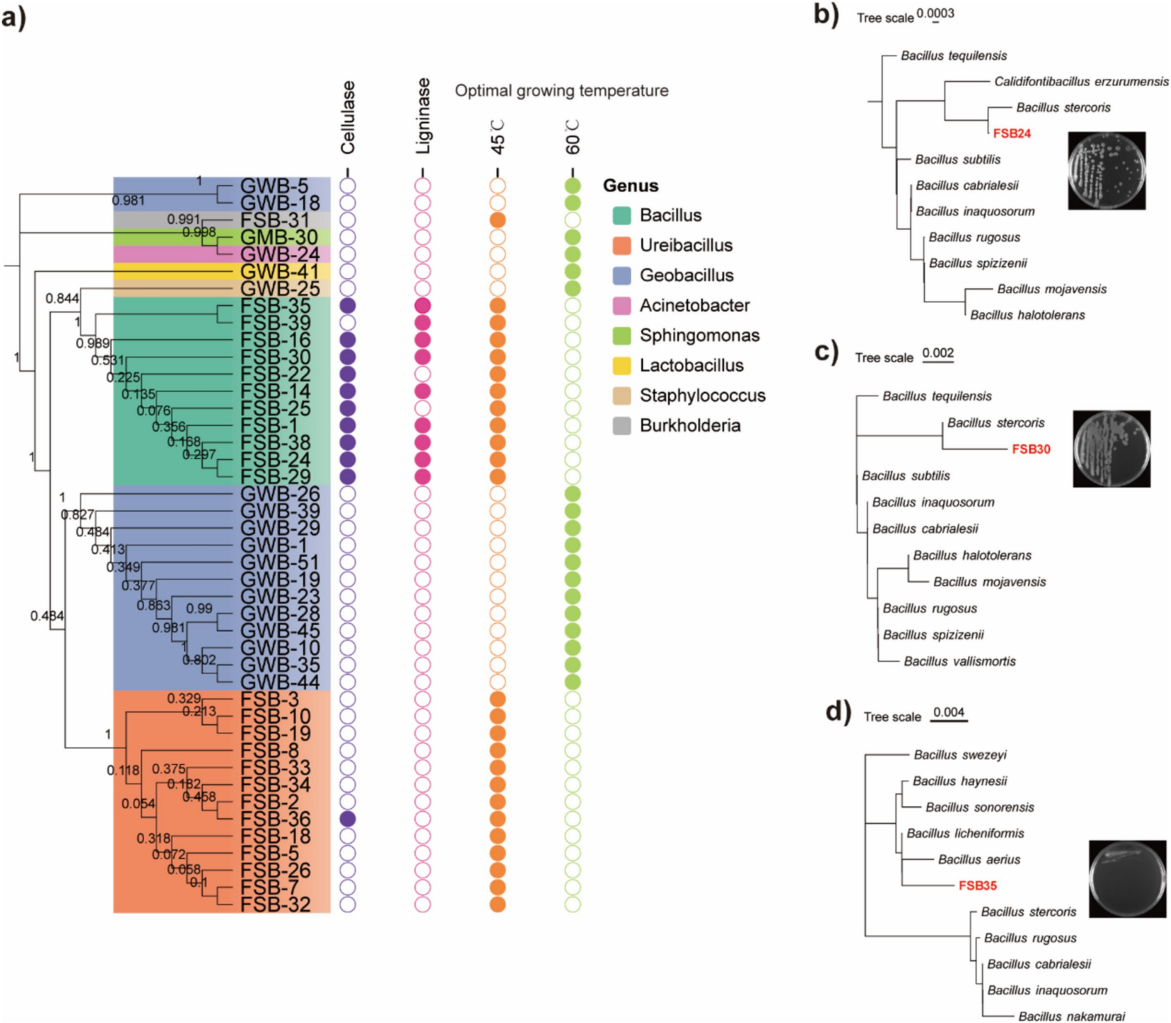
Thermophilic bacteria isolated through high-throughput culturing. **(a)** Phylogenetic tree of the 47 isolated bacterial strains assessed through 16S rDNA region using the maximum likelihood method and 1,000 bootstrap replicates in MEGA 7.0, their enzymatic production activities, and optimum incubation temperature. **(b)** Morphology and classification of isolate FSB24 assessed through 16S rDNA conserved region. **(c)** Morphology and classification of isolate FSB30 assessed through 16S rDNA conserved region. **(d)** Morphology and classification of isolate FSB35 assessed through 16S rDNA conserved region.

A total of 47 bacterial isolates were obtained from the plate culture based on colony morphology, color and shape. The 47 bacterial isolates were identified through 16S rDNA sequencing (Supplementary Table S3). A maximum likelihood dendrogram was generated with the 16S rDNA sequences of the bacteria and representative sequences from the databases. Phylogenetic analysis of the 47 isolates mainly matched with the genera of *Bacillus*, *Ureibacillus*, *Geobacillus*, *Acinetobacter*, *Sphingomonas*, *Lactobacillus*, *Staphylococcus*, and *Burkholderia*. Among the 47 bacterial isolates, 11 isolates produced the cellulose enzyme. A clear halo zone was found around the colonies in the Congo red agar plates (Figure 3a; Supplementary Figure S4). Nine of the isolates were able to produce lignin peroxidase, as clear halo zones were observed around the colonies in the aniline blue agar plates (Figure 3a; Supplementary Figure S5).

### Influence of microbial inoculum on manure compost maturity

**Figure 4:**
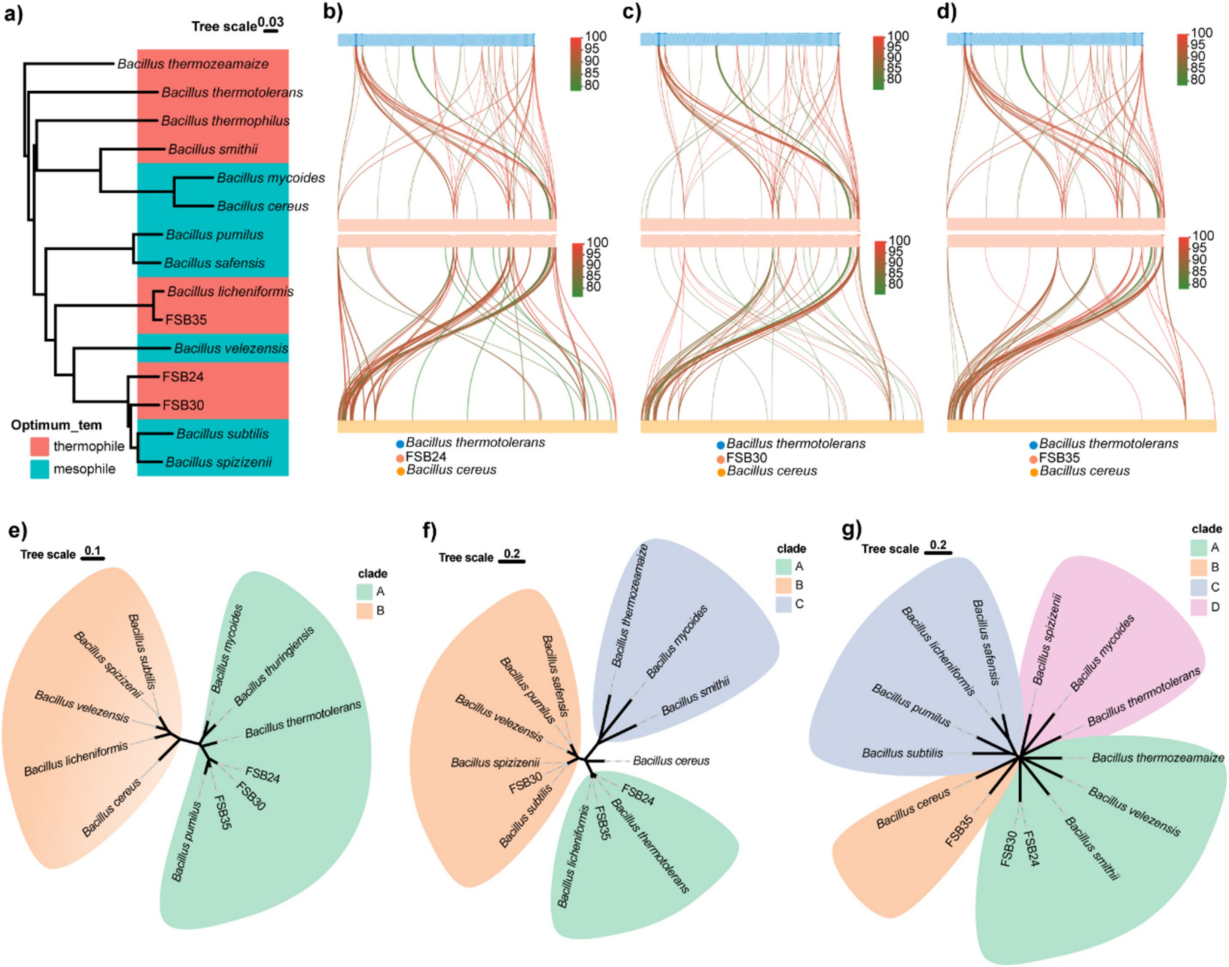
Genomic comparison of typical thermophiles and mesophiles in the genus of *Bacillus*. **(a)** Phylogenome analysis of the three isolates used in this study and 9 known *Bacillus* thermophiles and mesophiles were conducted by Mauve 2.4.0 and visualized through ggtree in R v4.3.2. **(b)** Genomic collinearity analysis of FSB24 with thermophile *Bacillus thermotolerans* and mosophile *Bacillus cereus*. **(c)** Genomic collinearity analysis of FSB30 with thermophile *B. thermotolerans* and mosophile *B. cereus*. **(d)** Genomic collinearity analysis of FSB35 with thermophile *B. thermotolerans* and mosophile *B. cereus*. **(e)** Phylogenetic analysis of small acid-soluble protein (SASP) sequences in various *Bacillus* species. **(f)** Phylogenetic analysis of fatty acid desaturase (DesE) sequences in various *Bacillus* species. **(g)** Phylogenetic analysis of transcription factor LysR sequences in various *Bacillus* species.

According to the 16S rDNA conserved region taxonomy identification and enzyme production, three isolates FSB24, FSB30, and FSB35, were selected as microbial inocula, and they were classified to *Bacillus stercoris*, *B. stercoris*, and *Bacillus licheniformis*, respectively, wherein *B. stercoris* is the sub-species of *Bacillus subtilis* (Figures 3b–d). In fact, through our subsequent whole genome sequencing, the taxonomy of FSB24 and FSB30 were closer to *Bacillus spizizenii* (Figure 4a).

**Figure 5:**
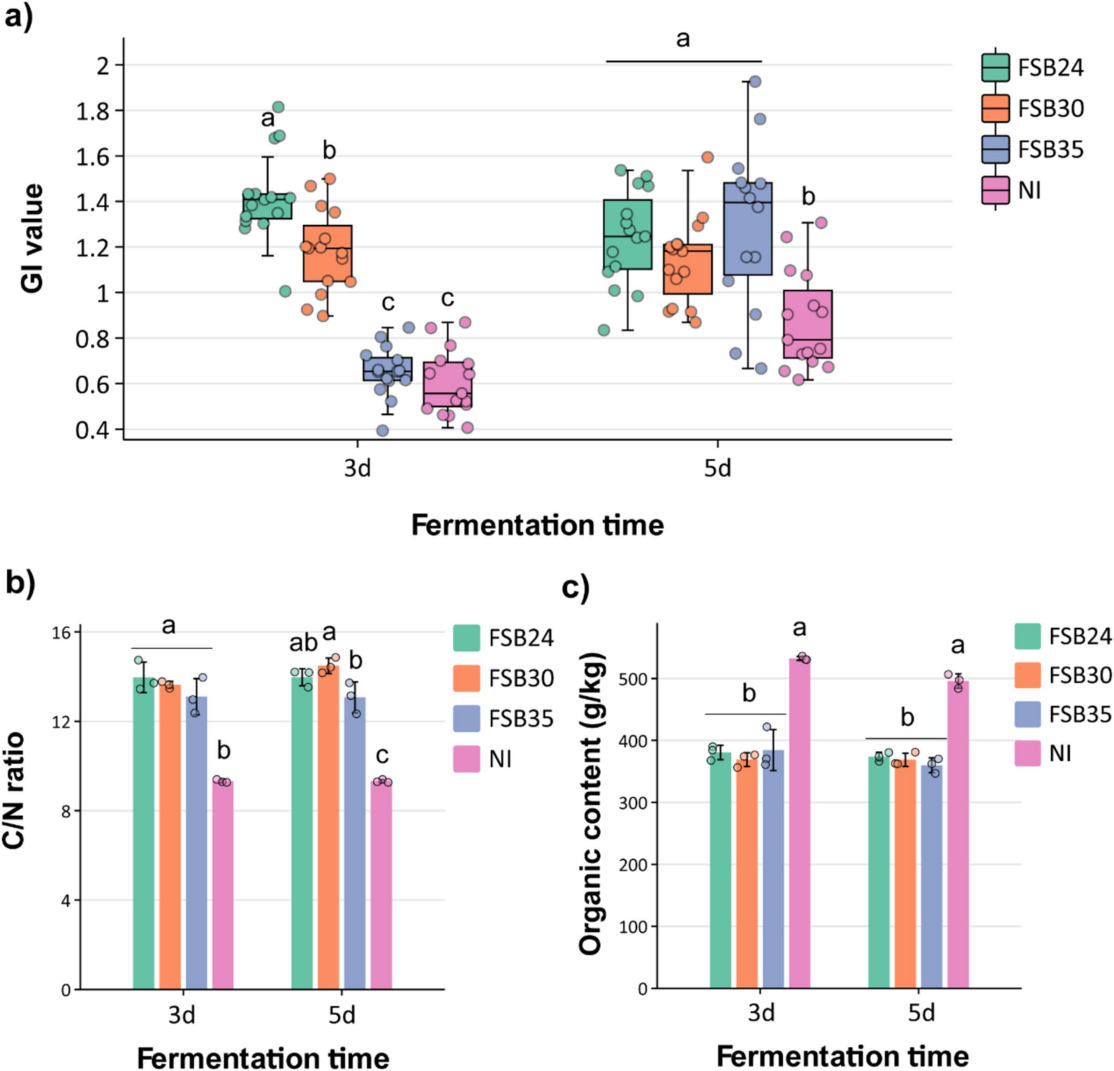
Influence of microbial inoculation on compost physiochemical characteristics after 3 days and 5 days of thermophilic composting. **(a)** Germination index (GI) index of the composts inoculated by the three bacterial isolates, individually. **(b)** C/N ratio of the composts inoculated by the three bacterial isolates, individually. **(c)** Organic content of the compost inoculated by the three bacterial isolates, individually. Bars indicate standard errors of the means. For a given duration of heat treatment, bars designated with the same letter indicate means that are not significantly different based on Fisher’s LSD analysis. NI: no inoculation.

Generally, compost is considered mature when the GI value of seed germination is >50%, and it is considered completely mature when GI is >80% (Zucconi et al., 1981). The compost inoculated with microbial agents FSB24 and FSB30 had GI values higher than 90% after 3 days of fermentation, which were significantly higher than those inoculated with FSB35 and no inoculation (NI) (Figure 5a). After 5 days of fermentation, the compost inoculated with microbial agents all possessed GI values significantly higher than the NI, among which the GI of FSB35 and NI were significantly increased comparing to 3 days’ fermentation (Figure 5a).

It is generally believed that compost is considered mature when its carbon to nitrogen (C/N) ratio close to 16, which is the C/N ratio of microorganisms (Li et al., 2022). The C/N ratios of compost inoculated with microbial agents FSB24, FSB30 and FSB35 after 3 days of fermentation were 13.97, 13.64, and 13.10 respectively, which were significantly higher than that of the NI (Figure 5b). The C/N values of compost inoculated with microbial inoculation after 5 days of fermentation were 13.97, 14.49, and 13.07, respectively, which were again significantly higher than that of the NI (Figure 5b). Additionally, the C/N ratios of FSB30 was closer to 16, indicating this treatment possessed higher degree of maturity than others (Figure 5b).

The initial organic matter content (OMC) of the raw manure materials was 54.38% (Supplementary Table S1). After fermentation, the OMC of all the treatments decreased, while the OMC of the composts with microbial inoculations were significantly lower than that of the NI regardless of the fermentation days, indicating that the microbial inoculation did a good job in decomposing the organic matter in the manure (Figure 5c). The OMC were not significantly different from each other among the three inoculation treatments regardless of the fermentation days (Figure 5c).

The total soluble phosphorus content of compost inoculated with microbial agents were all significantly higher than that of the NI after fermentation for 3 days. In addition, phosphorus content of the compost inoculated with FSB35 was significantly higher than that inoculated with FSB24 at 3 days of fermentation (Supplementary Figure S6a). At 5 days post fermentation, the phosphorus content of compost inoculated with microbial agents FSB35 was significantly higher than the NI, whereas the phosphorus content were not different among composts inoculated with FSB24, FSB30, and the NI (Supplementary Figure S6a).

The total potassium content of compost inoculated with microbial agents FSB35 was significantly higher than that inoculated with FSB24 after 3 days of fermentation, however, the potassium content in the composts inoculated with FSB24, FSB30 and the NI were not significantly different among each other (Supplementary Figure S6b). The total potassium content of compost after 5 days of fermentation were not significantly different among each other (Supplementary Figure S6b).

The conductivity of all the treatments were not significantly different among each other at 3 days of fermentation (Supplementary Figure S6c). However, the conductivity of the NI significantly decreased at 5 days of fermentation comparing to 3 days, resulting in a significantly lower value than the composts with microbial inoculations (Supplementary Figure S6c).

### Effect of fermented manure with microbial inoculation on wheat growth

**Figure 6:**
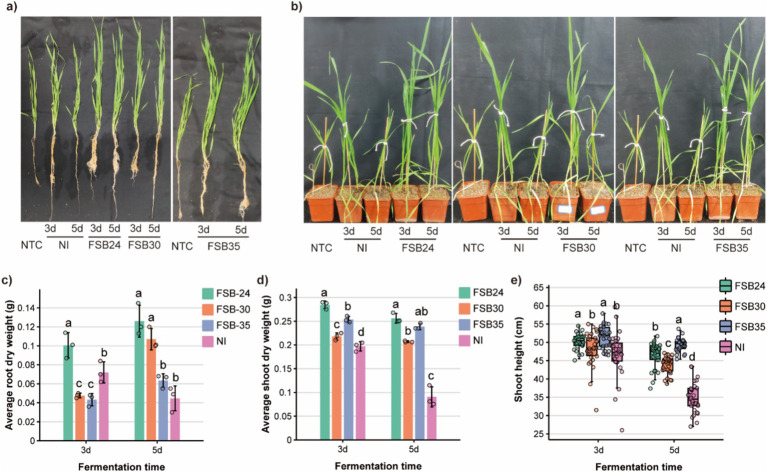
Quality of composts from microbe-aided thermophilic composting tested on wheat growth. **(a)** Root and shoot phenotypes of wheat fertilized by composts. **(b)** Phenotypes of wheat in pots fertilized by composts. **(c)** Root dry weight of wheat fertilized by composts treated with different microbial inoculum. **(d)** Shoot dry weight of wheat fertilized by composts treated with different microbial inoculum. **(e)** Shoot height of wheat fertilized by composts treated with different microbial inoculum.

Overall, the wheat cultivated in substrates mixed with composts grew better comparing to no treatment control (NTC) (Figures 6a,b). The root dry weight of wheat cultivated in 3-day-fermentation compost inoculated with microbial agents FSB24 was greater than other microbial inoculation treatments and the NI (Figure 6c). The root dry weight of wheat cultivated in 5-day-fermentation compost that inoculated with FSB24 and FSB30 were greater than those of the FSB35 and the NI treatments, whereas FSB35 and NI were not significantly different from each other (Figure 6c).

The shoot dry weight of wheat cultivated in 3-day-fermentation compost that inoculated with microbial agents were greater than that of the NI, and the wheat cultivated in compost inoculated with FSB24 performed the best in shoot growth (Figure 6d). Likewise, the shoot dry weight of wheat cultivated in 5-day-fermentation compost that inoculated with microbial agents were greater than that of the NI, and the wheat cultivated in compost inoculated with FSB24 performed significantly greater shoot growth than that of the FSB30 (Figure 6d).

The shoot height of wheat cultivated in 3-day-fermentation compost that inoculated with FSB24 and FSB35 were significantly greater than that of the FSB30 and NI (Figure 6e). The shoot height of wheat cultivated in 5-day-fermentation compost that inoculated with microbial agents were all significantly greater than that of the NI (Figure 6e). Wheat cultivated in 5-day-fermentation compost inoculated with FSB35 had the greatest shoot height among all the treatments (Figure 6e).

### Genome sequencing and comparative genomics

The genome sequence of the three bacteria generated about 1.2 Gb clean data, and the coverage base on reads mapping of FSB24, FSB30, and FSB35 were 98.70, 98.76, and 98.81%, respectively. Comparing to the genome of mesophilic bacterium *B. cereus*, the genome sizes of the three isolates, FSB24, FSB30, and FSB35 were smaller, but they all larger than the genomes of thermophilic bacterium *B. thermotolerans* (Table 1). The genomes of FSB24, FSB30, and FSB35 comprised of 8, 21, and 23 scaffolds, with GC contents of 43.38, 43.93, and 45.88%, respectively (Table 1), which were consistent with their (species) conformis genomes *B. spizizenii* (GCF_000227465.1) and *B. licheniformis* (GCF_000011645.1), but higher than the mesophilic bacterium *B. cereus* (Table 1).

**Table 1:** Genomic features of the three isolates used in this study and four other published *Bacillus* species.

Sample name	Genome size (Mb)	Total scarf no.	Scaffold N50 (Mb)	G + C (%)	Gene no.	Protein-coding
FSB24	4.04	8	2.13	43.38	4,560	4,292
FSB30	3.99	21	0.51	43.93	4,395	4,118
FSB35	4.14	23	2.12	45.88	4,832	4,482
*Bacillus spizizenii*	4.20	2	4.20	44.00	4,286	4,045
*Bacillus cereus*	5.40	1	5.00	35.50	5,497	5,255
*Bacillus licheniformis*	4.30	2	4.10	45.50	4,492	4,384
*Bacillus thermotolerans*	3.80	124	0.07	44.50	4,019	4,019

There were 4,292, 4,118, and 4,482 protein coding genes (CDS) recognized on the genomes of FSB24, FSB30, and FSB35, respectively, which were more than that on the conformis genomes of *B. spizizenii* and *B. licheniformis* as well as the thermophilic bacterium *B. thermotolerans* (Table 2). Whereas the numbers of CDS on the genome of the three isolates were smaller than the mesophilic bacterium *B. cereus* (Table 1). Among the three isolates, FSB30 and FSB35 comprised of plasmids (Table 2). Additionally, the tandem repeat number in isolates FSB24 and FSB30 were much lower than that in FSB35 (Table 2).

**Table 2:** Gene features of the three isolates used in this study.

Sample name	tRNAs no.	rRNAs no.	sRNA no.	Tandem repeat no.	Transposon no.	Plasmid no.
FSB24	80	9	92	18	4	0
FSB30	84	6	91	34	3	5
FSB35	79	11	96	81	3	8

**Figure 7:**
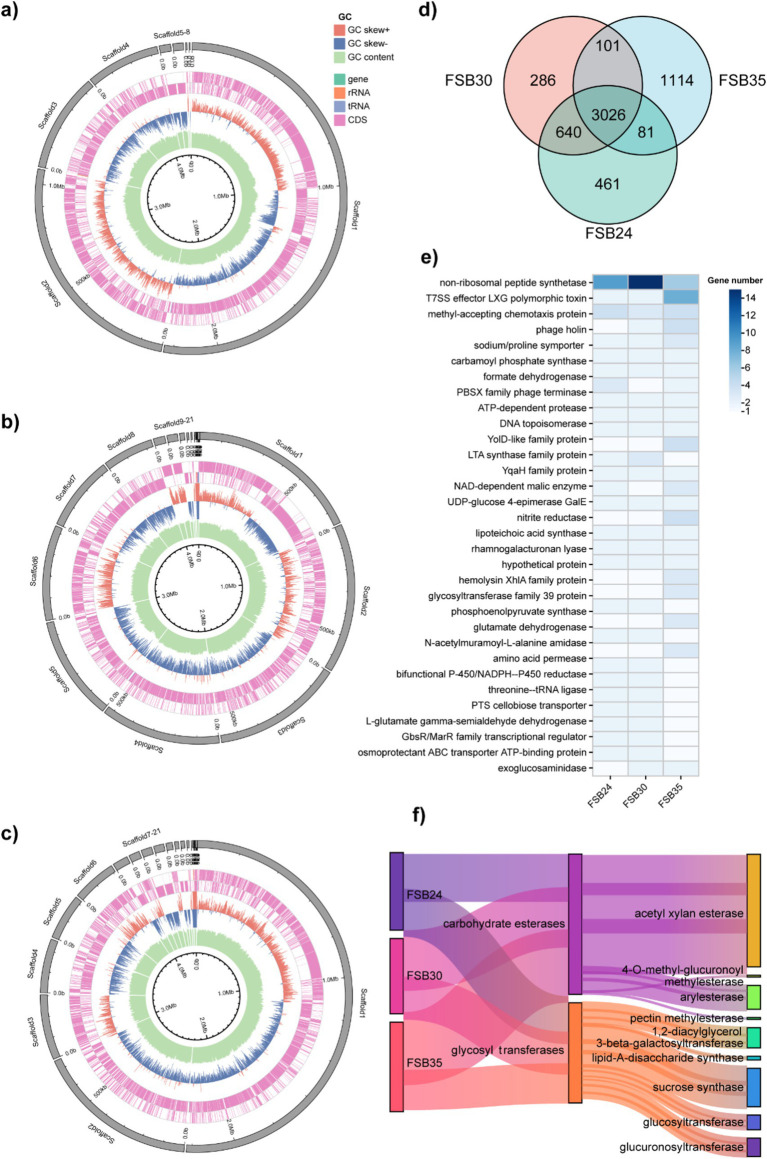
Comparative genomic analysis of the bacterial isolates FSB24, FSB30, and FSB35. **(a–c)** Circos display of the genomes of FSB24, FSB30, and FSB35, respectively. **(d)** Common and unique genes on the genome of FSB24, FSB30, and FSB35 as displayed through Venn diagram. **(e)** Homologous gene families with more than 5 homologous genes that obtained through comparing the genomes of FSB24, FSB30, and FSB35. **(f)** The abundance of two carbohydrate active enzyme classes, carbohydrate esterases and glycosyl transferases, on the genomes of FSB24, FSB30, and FSB35.

There were 3,026 genes commonly found in the three isolates, while there were 266, 461, and 1,114 unique genes found in FSB24, FSB30 and FSB35, respectively (Figure 7d). In total 3,932 homologous gene families were found through OrthoMCL annotation, including 33 of them possessed homologous genes ≥5. The 33 homologous gene families contain genes functioning in some basic life activities, such as non-ribosomal peptide synthesis, cell wall synthesis (LTA synthase family protein) and glycosyl transfer (Figure 7e). Besides, there are also homologous genes that explain why the three strains of bacteria are suitable for survival under stress (NADP-malic enzyme) (Chen et al., 2019). In addition, there are more than 5 homologous genes annotated to phosphotransferase system (PTS), which is a well-documented microbial system with a prominent role in carbohydrates transportation (Xu et al., 2023).

CAZymes play a significant role in degradation of complex carbohydrates. In the six categories of CAZymes, number of genes in carbohydrate esterases and glycosyl transferases presented obvious differences among the three isolates (Figure 7f). In carbohydrate esterases group, acetylxylan esterase plays an important role in the hydrolysis of xylan and possess 16, 20 and 22 genes in FSB24, FSB30 and FSB35, respectively (Figure 7f). Pectin methylesterase, which plays a critical role in modifying pectins, was only found in FSB35 (Figure 7f). In glycosyl transferases group, lipid-A-disaccharide synthase, which function in lipopolysaccharide biosynthesis, was only found in FSB24 and FSB35. The genes annotated to sucrose synthase in FSB24, FSB30, and FSB35 were 8, 6, and 7, respectively (Figure 7f).

Phylogenomics analysis of the three isolates together with 12 bacteria in the genus of *Bacillus* revealed that their genomes was classified by evolutionary relationships rather than heat tolerance (Figure 4a). Comparing to the genome of thermophilic bacterium *B. thermotolerans*, 7.45, 5.53, and 5.74% of genes on the genomes of FSB24, FSB30 and FSB35 were homologous (with identity ≥80%), respectively (Figures 4b–d).

Targeted to specific genes, we found that some genes in the three isolates homologous to *B. thermotolerans* were not homolog to mesophile *B. cereus*. The genes annotated to small acid-soluble protein (SASP) in the three isolates were more similar to *B. thermotolerans* according to the phylogenetic analysis than to the mesophiles *B. subtilis*, *B. velezensis*, *B. cereus* and their conformis genomes (Figure 4e). SASP are double-stranded DNA-binding proteins that contribute to the dormant spore’s high resistance to UV radiation by protecting the DNA backbone from enzymatic and chemical cleavage. The genes in FSB24 and FSB35 that encoded fatty acid desaturase were more similar to that in the thermophiles *B. thermotolerans* and *B. licheniformis* rather than the mesophiles (Figure 4f). Fatty acid desaturase catalyzes the desaturation reactions of saturated fatty acids thereby accelerate the metabolism of fatty acid. In addition, through phylogenetic analysis we found that transcriptional factor LysR of these three isolates were distantly away from their respective reference genomes but close to the thermophiles *B. thermozeamaize* and *B. smithii* (Figure 4g). LysR-type transcriptional regulators (LTTRs) regulate a diverse set of genes, including those involved in virulence, metabolism, quorum sensing and motility (Maddocks and Oyston, 2008).

## Discussion

Both thermophilic and lignocellulolytic microbe inoculations are cutting-edge strategies for expediting fecal fermentation and improve the production efficiency of organic fertilizer. Through 16S rRNA amplicon sequencing, we studied the microbial community evolution during livestock manure thermophilic composting. A clear microbial community alteration occurred mainly during the TC step. The abundance of thermotolerant bacterial species, such as *B. thermolactis,* significantly increased during composting, and correspondingly through function prediction, we found the abundance of cellulolysis and xylanolysis species were significantly higher in thermophilic process relative to the control. On the basis of the microbial succession and functional prediction, a total of 47 bacteria were *in situ* isolated and three of them with cellulose and lignin degradation ability were back inoculated to the manure, resulting in improved compost maturity and enhanced manure quality. Through comparative genomics analysis, it was demonstrated that, thermophilic feature is not a result of large-scale genomic alterations, but the changes in specific features and genes, such as the genome reduction, high GC content, and specific gene mutations.

The microbial dynamics in conventional manure waste composting has been well-studied (Chandrashekhar Parab and Prajapati, 2023; Wang et al., 2020), whereas the microbial community in the rapid thermophilic composting process that used in the present study is not very clear. Same as conventional aerobic composting, microbial community in rapid thermophilic composting has also undergone a succession from mesophiles-dominated to thermophiles-dominated, whereas the speed of community dynamic was faster and the community composition was more stable after TC step (Figures 1–3). For instance, though the HT stage in our fermentation system occurred within only 10 h, there have been significantly changes in *α* diversity (Figures 1a,b) and taxonomy abundance (Figures 1e,f) of the bacterial communities. High temperature is a kind of selection pressure, in which taxa with thermophilic or thermotolerant features become active as the process of composting continued, while those not well adapted to environmental changes either died out or entered a dormant state (Meng et al., 2019), resulting in a significantly simpler structure of the community with a lower total microbial diversity. Indeed, the α diversity of bacterial community was significantly decreased during the thermophilic step in the current study, which was in line with the findings of earlier researches (Xie et al., 2021; Cruz-Paredes et al., 2021). Additionally, we found the bacterial phylum of Firmicutes, which consist of the genus *Bacillus* with high tolerance to multiple stresses, was significantly increased in TC and CL steps (Figure 1e). These results suggested that the succession and formation of microbial communities can occur in a heterogeneous and targeted way due to niche differentiation and changes in the fundamental characteristics of composts, such as temperature and nutrient availability (Wei et al., 2018; Zhong et al., 2020).

In the current study, the microbe-aided thermophilic composting method required just 3 days to produce a mature and stable compost with the ideal addition of microbial inocula, which was much superior to the traditional composting, indicating that the inocula significantly shortened the time needed to produce a mature compost. There are two principal traits for sifting an effective composting microbial additive, thermotolerant and biomass degradation. Different composting piles were dominated by different microbial species, and each of which was adapted to a particular environmental state that varied during each composting stage (Zhao et al., 2018; Qiao et al., 2019; Wu et al., 2020). In a farm wastes and food wastes composting system, the prevalent bacteria were mainly from the genera of *Bacillus*, *Halobacillus* and *Staphylococcus* (Girish Chander et al., 2018), which was consistent with that of our rapid thermophilic composting. Species from the genus *Bacillus* have been reported to survive in various abiotic and biotic stresses through germination of spores, and possess the ability to secret kinds of enzymes. Therefore, they are often used in industry to produce highly active and high-purity amylase and protease (Gardener, 2004). Agro-industrial wastes generated from livestock manure consist of a large amount of animal proteins, as well as cellulose, hemicelluloses, and lignin originated from their feeds. Bacteria in the manure can promote refractory organic matter decomposition and nutrient transformation by increasing their own metabolic activity and extracellular enzyme production, and thus finally affect the compost maturity (Xie et al., 2021; Liu et al., 2023b). *Bacillus*, *Ureibacillus*, and *Geobacillus* are all in the family of Bacillaceae and reported to have various traits including N-fixation, P-solubilization, plant growth promotion, biological control and bio-fertilization (Singh et al., 2008; Richardson and Simpson, 2011; Sharma et al., 2013). Among the 47 bacteria isolated from TC and CL steps, 11 of them were in the genus of *Bacillus* in the current study (Figure 3a), and eight of them were able to degrade both cellulose and lignin (Supplementary Figures 4, 5).

Organisms that can live at temperature above 60°C are known as thermophiles. Research on the survival strategies of the thermophiles has gained a lot of attention because it sheds light on how life can thrive under extreme temperatures, and the potential applications in biotechnology. The majority of studies have concentrated on the characteristics of particular molecules, such as stability of protein structures (Bezsudnova et al., 2012) and/or the activity of thermophiles enzymes (Hakulinen et al., 2003). High temperature can no doubt induce genomic evolution, which in turn provides the bacteria with thermal-tolerant ability. Gene loss, gene mutations, or horizontal gene transfer (HGT) could all lead to such evolutionary alterations (Averhoff and Müller, 2010).

Comparative analysis on hundreds of genomes demonstrated that more than 20% of the bacterial genes and 40% of the archaeal genes are horizontally transferred (Gu and Hilser, 2009; Pang and Allemann, 2007). In the current study, we explored the common features among the three thermophile isolates that related to thermal adaptability. The random movement of transposon can create gene mutations that result in obtaining of new functions. In this study, the transposon helitron and long interspersed nuclear elements (LINE) were shared in all the three isolates, which was similar to geminiviruses, a virus that endemic to tropical and subtropical climates (Feschotte and Wessler, 2001). Besides, several homologous genes among the three isolates were reported to play a role in stress adaptation, such as the gene encoded NADP-malic enzyme. In plant, NADP-malic enzyme is essential to break down malate, which is necessary for maintaining the cytoplastic pH, regulating stomatal aperture, and boosting defenses against pathogens and excess aluminum (Chen et al., 2019).

The genome size of thermophiles are usually smaller than those of non-thermophiles (Wang et al., 2015). It was demonstrated that species that live at temperatures >60°C have genomes smaller than 4 Mb, whereas all species with genomes larger than 6 Mb live at temperatures lower than 45°C (van Noort et al., 2013). Likewise, in the current study, genome size of the three MI isolates were smaller than the mesophilic bacterium *B. cereus* and their reference genome *B. spizizenii* and *B. licheniformis*, but larger than the thermophile *B*. *thermotolerans* (Table 1). A possible explanation is that thermophiles used a cost-minimizing mechanism to adjust to external temperature changes by reducing the functional complexity of their genomes (Burra et al., 2010; Das et al., 2006). It is still up to dispute though, if thermophiles could delete genes that encode proteins with low thermo-stabilities during evolution (Sabath et al., 2013).

In addition to the changes in typical genomic features, the three MI isolates were genetically classified by their taxonomy, indicating that the acquisition of heat tolerance is not a global alteration on the genome, but a variation in specific heat-sensitive genes. Temperature is expected to dictate cell membrane lipid composition, such as fatty acid chain length and types of lipid headgroups, which in turn regulates the uptake and dissipation of ion gradients across biological membranes (Sollich et al., 2017). The mutation of *fabA* gene in *Escherichia coli* increased the degree of saturation in membrane lipids, resulting in enhanced adaptation to elevated temperatures (Blaby et al., 2012). In this study, the gene encoded fatty acid desaturase (*DesE*) in FSB24 and FSB35, which catalyzes the desaturation reactions of saturated fatty acids, were classified with those in thermophiles *B. thermotolerans* and *B. licheniformis* rather than the mesophiles (Figure 4f), suggesting the fatty acid saturation in the membrane of thermopiles may altered.

In recent years, due to the increase in conventional bedding material costs, an increasing number of farmers choose to use harmless recycled manure as bedding. Manure bedding treatment of farms can solve the problem of not only manure pollution, but also resource utilization. Under the decomposition of microorganisms, organic matter in manure is largely transferred into bio-available nitrogen, phosphorus, and potassium that can be absorbed by plants. In the current study, the addition of microbial inoculum has significant technical and economic advantages in promoting rapid composting, and shortening the fermentation cycles (Figures 5, 6). We observed significant decreasing of organic matter content in compost with microbial inoculation relative to NI, indicating inocula played an important role in consuming the organic matter in manure (Figure 5c). In addition, the growth-promoting effect of compost inoculated with microbial inoculation were highly improved relative to the compost without inoculation (Figure 6). Intriguingly, in our study, FSB35 (*B. licheniformis*) did a better job in releasing bio-available phosphorus and potassium comparing to the other two isolates (*B. spizizenii*) (Supplementary Figures 6a,b). *B. licheniformis* has been regarded as an outstanding microbial cell factory for the production of biochemicals and enzymes (Zhan et al., 2020), including cellulose, hemicellulose and various thermo-tolerant proteinases, and it is also used as a plant growth promoter (Nunes et al., 2023), thus has widely application value.

## Conclusion

Different from the conventional aerobic composting, the composting process in a bio-reactor is more rapid and efficient. Mimicking the conditions of tank composting, we found that the bacterial community has also undergone a succession from mesophiles-dominated to thermophiles-dominated, but the community changed at an accelerated pace, with a decline in diversity and a trend toward simplified structure. Majority of the bacteria isolated through high throughput cultivation method were identified as *Bacillus*, *Ureibacillus*, and *Geobacillus*, which are in the phylum of Firmicutes. Three *Bacillus* isolates with cellulose and lignin degradation ability were back inoculated to the manure, resulting in 3-day fast maturity and improved compost quality as fertilizers, especially in terms of promoting wheat growth. Comparing to the genomes of mesophilic and thermophilic *Bacillus*, the genomes of the three isolates manifested some features closer to the thermophiles, not only including the typical genomic features such as shrunken genome size, but also particular genes’ mutation that related to heat tolerance, such as membrane saturation. The current study indicated that in the microbe-aided thermophilic composting system, the improvement of composting efficiency was due to the prevalence of thermophiles with specific functions. This study has crucial implications for the resource utilization of livestock manure.

## Data Availability

The datasets presented in this study can be found in online repositories. The names of the repository/repositories and accession number(s) can be found in the article/Supplementary material.

## Footnotes

^1^http://ieg2.ou.edu/MENA

^2^https://github.com/tseemann/barrnap

^3^http://geneontology.org/

^4^http://www.genome.jp/kegg/

^5^http://www.ncbi.nlm.nih.gov/COG/

^6^http://www.ebi.ac.uk/uniprot/

## References

[ref1] Averhoff B. Müller V. (2010). Exploring research frontiers in microbiology: recent advances in halophilic and thermophilic extremophiles. Res. Microbiol. 161, 506–514. doi: 10.1016/j.resmic.2010.05.006 20594981

[ref2] Benson G. (1999). Tandem repeats finder: a program to analyze DNA sequences. Nucleic Acids Res. 27, 573–580. doi: 10.1093/nar/27.2.573, PMID: 9862982 PMC148217

[ref3] Bezsudnova E. Y. Boyko K. M. Polyakov K. M. Dorovatovskiy P. V. Stekhanova T. N. Gumerov V. M.. (2012). Structural insight into the molecular basis of polyextremophilicity of short-chain alcohol dehydrogenase from the hyperthermophilic archaeon. Biochimie 94, 2628–2638. doi: 10.1016/j.biochi.2012.07.024, PMID: 22885278

[ref4] Blaby I. K. Lyons B. J. Wroclawska-Hughes E. Phillips G. C. F. Pyle T. P. Chamberlin S. G.. (2012). Experimental evolution of a facultative thermophile from a mesophilic ancestor. Appl. Environ. Microb. 78, 144–155. doi: 10.1128/Aem.05773-11, PMID: 22020511 PMC3255606

[ref5] Blin K. Medema M. H. Kazempour D. Fischbach M. A. Breitling R. Takano E.. (2013). Anti SMASH 2.0-a versatile platform for genome mining of secondary metabolite producers. Nucleic Acids Res. 41, W204–W212. doi: 10.1093/nar/gkt449, PMID: 23737449 PMC3692088

[ref6] Buchfink B. Reuter K. Drost H. G. (2021). Sensitive protein alignments at tree-of-life scale using DIAMOND. Nat. Methods 18, 366–368. doi: 10.1038/s41592-021-01101-x, PMID: 33828273 PMC8026399

[ref7] Burnley S. Phillips R. Coleman T. Rampling T. (2011). Energy implications of the thermal recovery of biodegradable municipal waste materials in the United Kingdom. Waste Manag. 31, 1949–1959. doi: 10.1016/j.wasman.2011.04.015, PMID: 21600755

[ref8] Burra P. V. Kalmar L. Tompa P. (2010). Reduction in structural disorder and functional complexity in the thermal adaptation of prokaryotes. PLoS One 5:e12069. doi: 10.1371/journal.pone.0012069, PMID: 20711457 PMC2920320

[ref9] Chan P. P. Lowe T. M. (2019). tRNAscan-SE: searching for tRNA genes in genomic sequences. Methods Mol. Biol. 1962, 1–14. doi: 10.1007/978-1-4939-9173-0_1, PMID: 31020551 PMC6768409

[ref10] Chandrashekhar Parab K. D. Y. Prajapati V. (2023). Genomics and microbial dynamics in green waste composting: a mini review. Ecol. Genet. Genom. 29:100206. doi: 10.1016/j.egg.2023.100206

[ref11] Chaurasia A. Meena B. R. Tripathi A. N. Pandey K. K. Rai A. B. Singh B. (2018). Actinomycetes: an unexplored microorganisms for plant growth promotion and biocontrol in vegetable crops. World J. Microb. Biot. 34:132. doi: 10.1007/s11274-018-2517-5, PMID: 30105532

[ref12] Chen Q. Q. Wang B. P. Ding H. Y. Zhang J. Li S. C. (2019). Review: the role of NADP-malic enzyme in plants under stress. Plant Sci. 281, 206–212. doi: 10.1016/j.plantsci.2019.01.010, PMID: 30824053

[ref13] Chen S. F. Zhou Y. Q. Chen Y. R. Gu J. (2018). Fastp: an ultra-fast all-in-one FASTQ preprocessor. Bioinformatics 34, i884–i890. doi: 10.1093/bioinformatics/bty560, PMID: 30423086 PMC6129281

[ref14] Collins M. D. Hutson R. A. Falsen E. Sjödén B. (1999). *Facklamia tabacinasalis* sp. nov., from powdered tobacco. Int. J. Syst. Bacteriol. 49, 1247–1250. doi: 10.1099/00207713-49-3-1247, PMID: 10425787

[ref15] Coorevits A. Logan N. A. Dinsdale A. E. Halket G. Scheldeman P. Heyndrickx M.. (2011). *Bacillus thermolactis* sp. nov., isolated from dairy farms, and emended description of *Bacillus thermoamylovorans*. Int. J. Syst. Evol. Micr. 61, 1954–1961. doi: 10.1099/ijs.0.024240-0 20833876

[ref16] Cruz-Paredes C. Tájmel D. Rousk J. (2021). Can moisture affect temperature dependences of microbial growth and respiration? Soil Biol. Biochem. 156:108223. doi: 10.1016/j.soilbio.2021.108223

[ref17] Darling A. C. E. Mau B. Blattner F. R. Perna N. T. (2004). Mauve: multiple alignment of conserved genomic sequence with rearrangements. Genome Res. 14, 1394–1403. doi: 10.1101/gr.2289704, PMID: 15231754 PMC442156

[ref18] Das S. Paul S. Bag S. K. Dutta C. (2006). Analysis of Nanoarchaeum equitans genome and proteome composition: indications for hyperthermophilic and parasitic adaptation. BMC Genomics 7:186. doi: 10.1186/1471-2164-7-186, PMID: 16869956 PMC1574309

[ref19] De la Cruz F. B. Cheng Q. W. Call D. F. Barlaz M. A. (2021). Evidence of thermophilic waste decomposition at a landfill exhibiting elevated temperature regions. Waste Manag. 124, 26–35. doi: 10.1016/j.wasman.2021.01.014, PMID: 33596536

[ref20] Delcher A. L. Bratke K. A. Powers E. C. Salzberg S. L. (2007). Identifying bacterial genes and endosymbiont DNA with glimmer. Bioinformatics 23, 673–679. doi: 10.1093/bioinformatics/btm009, PMID: 17237039 PMC2387122

[ref21] Deng Y. Jiang Y. H. Yang Y. F. He Z. L. Luo F. Zhou J. Z. (2012). Molecular ecological network analyses. BMC Bioinf. 13. doi: 10.1186/1471-2105-13-113, PMID: 22646978 PMC3428680

[ref22] Dong W. Zhou R. Li X. Yan H. Zheng J. Peng N.. (2023). Effect of simplified inoculum agent on performance and microbiome during cow manure-composting at industrial-scale. Bioresour. Technol. 393:130097. doi: 10.1016/j.biortech.2023.130097, PMID: 38013035

[ref23] Edgar R. C. (2013). UPARSE: highly accurate OTU sequences from microbial amplicon reads. Nat. Methods 10, 996–998. doi: 10.1038/Nmeth.2604, PMID: 23955772

[ref24] Feschotte C. Wessler S. R. (2001). Treasures in the attic: rolling circle transposons discovered in eukaryotic genomes. P. Natl. Acad. Sci. U. S. A. 98, 8923–8924. doi: 10.1073/pnas.171326198, PMID: 11481459 PMC55346

[ref25] Gardener B. B. M. (2004). Ecology of Bacillus and *Paenibacillus* spp. in agricultural systems. Phytopathology 94, 1252–1258. doi: 10.1094/Phyto.2004.94.11.1252, PMID: 18944463

[ref26] Girish Chander S. P. W. Gopalakrishnan S. Mahapatra A. Swati Chaudhury C. S. Pawar M. K. Rao A. V. R. K. (2018). Microbial consortium culture and vermi-composting technologies for recycling on-farm wastes and food production. Int. J. Recycl. Org. Waste Agricult. 7, 99–108. doi: 10.1007/s40093-018-0195-9

[ref27] Gu J. Hilser V. J. (2009). Sequence-based analysis of protein energy landscapes reveals nonuniform thermal adaptation within the proteome. Mol. Biol. Evol. 26, 2217–2227. doi: 10.1093/molbev/msp140, PMID: 19592668 PMC2912464

[ref28] Gu Z. Q. Yan H. B. Zhang Q. Wang Y. P. Liu C. X. Cui X.. (2024). Elimination of copper obstacle factor in anaerobic digestion effluent for value-added utilization: performance and resistance mechanisms of indigenous bacterial consortium. Water Res. 252:121217. doi: 10.1016/j.watres.2024.121217 38335748

[ref29] Hakulinen N. Turunen O. Jänis J. Leisola M. Rouvinen J. (2003). Three-dimensional structures of thermophilic β-1,4-xylanases from *Chaetomium thermophilum* and *Nonomuraea flexuosa*: comparison of twelve xylanases in relation to their thermal stability. Eur. J. Biochem. 270, 1399–1412. doi: 10.1046/j.1432-1033.2003.03496.x, PMID: 12653995

[ref30] Hyatt D. Chen G. L. LoCascio P. F. Land M. L. Larimer F. W. Hauser L. J. (2010). Prodigal: prokaryotic gene recognition and translation initiation site identification. BMC Bioinf. 11:119. doi: 10.1186/1471-2105-11-119, PMID: 20211023 PMC2848648

[ref31] Jia X. B. Lin X. J. Tian Y. D. Chen J. C. You M. S. (2017). High production, purification, biochemical characterization and gene analysis of a novel catalase from the thermophilic bacterium FZSF03. Int. J. Biol. Macromol. 103, 89–98. doi: 10.1016/j.ijbiomac.2017.05.034, PMID: 28501604

[ref32] Jurak E. Punt A. M. Arts W. Kabel M. A. Gruppen H. (2015). Fate of carbohydrates and lignin during composting and mycelium growth of *Agaricus bisporus* on wheat straw based compost. PLoS One 10:e0138909. doi: 10.1371/journal.pone.0138909 26436656 PMC4593547

[ref33] Khalil A. Domeizel M. Prudent P. (2008). Monitoring of green waste composting process based on redox potential. Bioresour. Technol. 99, 6037–6045. doi: 10.1016/j.biortech.2007.11.043, PMID: 18178428

[ref34] Kristich C. J. Rice L. B. Arias C. A. (2014). “Enterococcal infection-treatment and antibiotic resistance” in Enterococci: From commensals to leading causes of drug resistant infection. eds. Gilmore M. S. Clewell D. B. Ike Y. Shankar N. (Boston, MA: Elsevier). 24649510

[ref35] Kumar S. Stecher G. Tamura K. (2016). MEGA7: molecular evolutionary genetics analysis version 7.0 for bigger datasets. Mol. Biol. Evol. 33, 1870–1874. doi: 10.1093/molbev/msw054, PMID: 27004904 PMC8210823

[ref36] Li L. Stoeckert C. J. Roos D. S. (2003). OrthoMCL: identification of ortholog groups for eukaryotic genomes. Genome Res. 13, 2178–2189. doi: 10.1101/gr.1224503, PMID: 12952885 PMC403725

[ref37] Li D. Y. Yuan J. Ding J. T. Wang H. H. Shen Y. J. Li G. X. (2022). Effects of carbon/nitrogen ratio and aeration rate on the sheep manure composting process and associated gaseous emissions. J. Environ. Manag. 323:116093. doi: 10.1016/j.jenvman.2022.116093, PMID: 36095985

[ref38] Lin X. Wang N. Y. Li F. H. Yan B. H. Pan J. T. Jiang S. L.. (2022). Evaluation of the synergistic effects of biochar and biogas residue on CO_2_ and CH_4_ emission, functional genes, and enzyme activity during straw composting. Bioresour. Technol. 360:127608. doi: 10.1016/j.biortech.2022.127608, PMID: 35840030

[ref39] Liu X. Rong X. Yang J. Li H. Hu W. Yang Y.. (2023b). Community succession of microbial populations related to CNPS biological transformations regulates product maturity during cow-manure-driven composting. Bioresour. Technol. 369:128493. doi: 10.1016/j.biortech.2022.128493, PMID: 36526118

[ref40] Liu G. L. Yang Y. Ma R. N. Jiang J. H. Li G. X. Wang J. N.. (2023a). Thermophilic compost inoculating promoted the maturity and mature compost inoculating reduced the gaseous emissions during co-composting of kitchen waste and pig manure. Environ. Technol. Inno. 32:103427. doi: 10.1016/j.eti.2023.103427

[ref41] Lomsadze A. Gemayel K. Tang S. Y. Y. Borodovsky M. (2018). Modeling leaderless transcription and atypical genes results in more accurate gene prediction in prokaryotes. Genome Res. 28, 1079–1089. doi: 10.1101/gr.230615.117, PMID: 29773659 PMC6028130

[ref42] Luo R. B. Liu B. H. Xie Y. L. Li Z. Y. Huang W. H. Yuan J. Y.. (2012). SOAPdenovo2: an empirically improved memory-efficient short-read assembler. Gigascience 1:18. doi: 10.1186/2047-217x-1-18 23587118 PMC3626529

[ref43] Ma F. Zhu T. Yao S. Quan H. Y. Zhang K. Liang B. R.. (2022). Coupling effect of high temperature and thermophilic bacteria indirectly accelerates the humification process of municipal sludge in hyperthermophilic composting. Process Saf. Environ. 166, 469–477. doi: 10.1016/j.psep.2022.08.052

[ref44] Maddocks S. E. Oyston P. C. F. (2008). Structure and function of the LysR-type transcriptional regulator (LTTR) family proteins. Microbiol. SGM 154, 3609–3623. doi: 10.1099/mic.0.2008/022772-0 19047729

[ref45] Magoc T. Salzberg S. L. (2011). FLASH: fast length adjustment of short reads to improve genome assemblies. Bioinformatics 27, 2957–2963. doi: 10.1093/bioinformatics/btr507, PMID: 21903629 PMC3198573

[ref46] Meng Q. X. Yang W. Men M. Q. Bello A. Xu X. H. Xu B. S.. (2019). Microbial community succession and response to environmental variables during cow manure and corn straw composting. Front. Microbiol. 10:529. doi: 10.3389/fmicb.2019.00529, PMID: 30936861 PMC6431636

[ref47] Niu K. F. Chao C. Zhang X. X. An Z. G. Zhou J. Y. Yang L. G. (2022). Effects of different microbial agents on bedding treatment of ectopic fermentation of buffalo manure. Front. Microbiol. 13:1080650. doi: 10.3389/fmicb.2022.1080650, PMID: 36620065 PMC9814712

[ref48] Nunes P. S. D. de Medeiros F. H. de Oliveira T. S. Zago J. R. D. Bettiol W. (2023). Bacillus subtilis and *Bacillus licheniformis* promote tomato growth. Braz. J. Microbiol. 54, 397–406. doi: 10.1007/s42770-022-00874-3, PMID: 36422850 PMC9943921

[ref49] Pajura R. (2023). Composting municipal solid waste and animal manure in response to the current fertilizer crisis - a recent review. Sci. Total Environ. 912:169221. doi: 10.1016/j.scitotenv.2023.169221 38101643

[ref50] Pang J. Y. Allemann R. K. (2007). Molecular dynamics simulation of thermal unfolding of *Thermatoga maritima* DHFR. Phys. Chem. Chem. Phys. 9, 711–718. doi: 10.1039/b611210b, PMID: 17268682

[ref51] Park S. Y. Kwon H. Kim S. G. Park S. C. Kim J. H. Seo S. (2023). Characterization of two lytic bacteriophages, infecting complex (SBSEC) from Korean ruminant. Sci. Rep. 13:9110. doi: 10.1038/s41598-023-36306-x, PMID: 37277552 PMC10241823

[ref52] Qiao C. C. Penton C. R. Liu C. Shen Z. Z. Ou Y. N. Liu Z. Y.. (2019). Key extracellular enzymes triggered high-efficiency composting associated with bacterial community succession. Bioresour. Technol. 288:121576. doi: 10.1016/j.biortech.2019.121576 31176934

[ref53] Rasmussen M. (2016). Aerococcus: an increasingly acknowledged human pathogen. Clin. Microbiol. Infec. 22, 22–27. doi: 10.1016/j.cmi.2015.09.026, PMID: 26454061

[ref54] Richardson A. E. Simpson R. J. (2011). Soil microorganisms mediating phosphorus availability. Plant Physiol. 156, 989–996. doi: 10.1104/pp.111.175448, PMID: 21606316 PMC3135950

[ref55] Sabath N. Ferrada E. Barve A. Wagner A. (2013). Growth temperature and genome size in bacteria are negatively correlated, suggesting genomic dtreamlining during thermal adaptation. Genome Biol. Evol. 5, 966–977. doi: 10.1093/gbe/evt050, PMID: 23563968 PMC3673621

[ref56] Sakai M. Deguchi D. Hosoda A. Kawauchi T. Ikenaga M. (2015). *Ammoniibacillus agariperforans* gen. Nov., sp. nov., a thermophilic, agar-degrading bacterium isolated from compost. Int. J. Syst. Evol. Micr. 65, 570–577. doi: 10.1099/ijs.0.067843-0, PMID: 25404482

[ref57] Sharma A. Shankhdhar D. Shankhdhar S. C. (2013). Enhancing grain iron content of rice by the application of plant growth promoting rhizobacteria. Plant Soil Environ. 59, 89–94. doi: 10.17221/683/2012-Pse

[ref58] Singh N. Pandey P. Dubey R. C. Maheshwari D. K. (2008). Biological control of root rot fungus *Macrophomina phaseolina* and growth enhancement of *Pinus roxburghii* (Sarg.) by rhizosphere competent *Bacillus subtilis* BN1. World J. Microb. Biot. 24, 1669–1679. doi: 10.1007/s11274-008-9680-z

[ref59] Sollich M. Yoshinaga M. Y. Häusler S. Price R. E. Hinrichs K. U. Bühring S. I. (2017). Heat stress dictates microbial lipid composition along a thermal gradient in marine sediments. Front. Microbiol. 8:1550. doi: 10.3389/fmicb.2017.01550, PMID: 28878741 PMC5572230

[ref60] Song C. H. Li M. X. Wei Z. M. Jia X. Xi B. D. Liu D. M.. (2016). Effect of inoculation with multiple composite microorganisms on characteristics of humic fractions and bacterial community structure during biogas residue and livestock manure co-composting. J. Chem. Technol. Biot. 91, 155–164. doi: 10.1002/jctb.4554

[ref61] Sun B. Zhang L. X. Yang L. Z. Zhang F. S. Norse D. Zhu Z. L. (2012). Agricultural non-point source pollution in China: causes and mitigation measures. Ambio 41, 370–379. doi: 10.1007/s13280-012-0249-6, PMID: 22311715 PMC3393061

[ref62] Teather R. M. Wood P. J. (1982). Use of Congo red polysaccharide interactions in enumeration and characterization of cellulolytic bacteria from the bovine rumen. Appl. Environ. Microb. 43, 777–780. doi: 10.1128/Aem.43.4.777-780.1982, PMID: 7081984 PMC241917

[ref63] Thompson J. D. Higgins D. G. Gibson T. J. (1994). Clustal-W-improving the sensitivity of progressive multiple sequence alignment through sequence weighting, position-specific gap penalties and weight matrix choice. Nucleic Acids Res. 22, 4673–4680. doi: 10.1093/nar/22.22.4673, PMID: 7984417 PMC308517

[ref64] van Noort V. Bradatsch B. Arumugam M. Amlacher S. Bange G. Creevey C.. (2013). Consistent mutational paths predict eukaryotic thermostability. BMC Evol. Biol. 13:7. doi: 10.1186/1471-2148-13-7, PMID: 23305080 PMC3546890

[ref65] Vlaskin M. S. Vladimirov G. N. (2018). Hydrothermal carbonization of organic components from municipal solid waste. Theor. Found. Chem. Eng. 52, 996–1003. doi: 10.1134/S0040579518050421

[ref66] Wainaina S. Awasthi M. K. Sarsaiya S. Chen H. Y. Singh E. Kumar A.. (2020). Resource recovery and circular economy from organic solid waste using aerobic and anaerobic digestion technologies. Bioresour. Technol. 301:122778. doi: 10.1016/j.biortech.2020.122778 31983580

[ref67] Wang Q. H. Cen Z. Zhao J. J. (2015). The survival mechanisms of thermophiles at high temperatures: an angle of omics. Physiology 30, 97–106. doi: 10.1152/physiol.00066.2013, PMID: 25729055

[ref68] Wang Q. Garrity G. M. Tiedje J. M. Cole J. R. (2007). Naive Bayesian classifier for rapid assignment of rRNA sequences into the new bacterial taxonomy. Appl. Environ. Microb. 73, 5261–5267. doi: 10.1128/Aem.00062-07, PMID: 17586664 PMC1950982

[ref69] Wang W. K. Liang C. M. (2021). Enhancing the compost maturation of swine manure and rice straw by applying bioaugmentation. Sci. Rep. 11:6103. doi: 10.1038/s41598-021-85615-6, PMID: 33731751 PMC7971061

[ref70] Wang T. T. Sun Z. Y. Wang S. P. Tang Y. Q. Kida K. (2020). Succession of total and active microbial community during the composting of anaerobic digested residue. Waste Biomass Valori 11, 4677–4689. doi: 10.1007/s12649-019-00779-7

[ref71] Wang L. Q. Zhao Y. Xie L. A. Zhang G. G. Wei Z. M. Li J.. (2023). The dominant role of cooperation in fungal community drives the humification process of chicken manure composting under addition of regulatory factors. Environ. Res. 232:116358. doi: 10.1016/j.envres.2023.116358 37295586

[ref72] Wei H. W. Wang L. H. Hassan M. Xie B. (2018). Succession of the functional microbial communities and the metabolic functions in maize straw composting process. Bioresour. Technol. 256, 333–341. doi: 10.1016/j.biortech.2018.02.050 29459320

[ref73] Wu N. Xie S. Y. Zeng M. Xu X. Y. Li Y. Liu X. Y.. (2020). Impacts of pile temperature on antibiotic resistance, metal resistance and microbial community during swine manure composting. Sci. Total Environ. 744:140920. doi: 10.1016/j.scitotenv.2020.140920, PMID: 32711322

[ref74] Xie G. X. Kong X. L. Kang J. L. Su N. Luo G. W. Fei J. C. (2021). Community-level dormancy potential regulates bacterial beta-diversity succession during the co-composting of manure and crop residues. Sci. Total Environ. 772:145506. doi: 10.1016/j.scitotenv.2021.145506 33571759

[ref75] Xu T. Tao X. Y. He H. X. Kempher M. L. Zhang S. P. Liu X. C.. (2023). Functional and structural diversification of incomplete phosphotransferase system in cellulose-degrading clostridia. ISME J. 17, 823–835. doi: 10.1038/s41396-023-01392-2, PMID: 36899058 PMC10203250

[ref76] Yan H. B. Gu Z. Q. Zhang Q. Wang Y. P. Cui X. Liu Y. H.. (2024). Detoxification of copper and zinc from anaerobic digestate effluent by indigenous bacteria: mechanisms, pathways and metagenomic analysis. J. Hazard. Mater. 469:133993. doi: 10.1016/j.jhazmat.2024.133993, PMID: 38461661

[ref77] Yang G. Q. Chen J. H. Zhou S. G. (2015). *Novibacillus thermophilus* gen. Nov., sp. nov., a gram-staining-negative and moderately thermophilic member of the family Thermoactinomycetaceae. Int. J. Syst. Evol. Micr. 65, 2591–2597. doi: 10.1099/ijs.0.000306 25951858

[ref78] Yang G. Q. Zhou S. G. (2014). *Sinibacillus soli* gen. Nov., sp. nov., a moderately thermotolerant member of the family Bacillaceae. Int. J. Syst. Evol. Micr. 64, 1647–1653. doi: 10.1099/ijs.0.055608-0, PMID: 24510979

[ref79] Zaman B. Hardyanti N. Purwono R. B. S. (2022). An innovative thermal composter to accelerate food waste decomposition at the household level. Biores. Technol. Rep. 19:101203. doi: 10.1016/j.biteb.2022.101203

[ref80] Zhan Y. Y. Xu Y. Zheng P. L. He M. Sun S. H. Wang D.. (2020). Establishment and application of multiplexed CRISPR interference system in *Bacillus licheniformis*. Appl. Microbiol. Biot. 104, 391–403. doi: 10.1007/s00253-019-10230-5, PMID: 31745574

[ref81] Zhang L. Sun X. Y. Tian Y. Gong X. Q. (2013). Effects of brown sugar and calcium superphosphate on the secondary fermentation of green waste. Bioresour. Technol. 131, 68–75. doi: 10.1016/j.biortech.2012.10.059, PMID: 23340104

[ref82] Zhao Y. Liu Z. Zhang B. Cai J. Yao X. Zhang M.. (2023). Inter-bacterial mutualism promoted by public goods in a system characterized by deterministic temperature variation. Nat. Commun. 14:5394. doi: 10.1038/s41467-023-41224-7, PMID: 37669961 PMC10480208

[ref83] Zhao X. Y. Wei Y. Q. Fan Y. Y. Zhang F. Tan W. B. He X. S.. (2018). Roles of bacterial community in the transformation of dissolved organic matter for the stability and safety of material during sludge composting. Bioresour. Technol. 267, 378–385. doi: 10.1016/j.biortech.2018.07.060 30031276

[ref84] Zhong X. Z. Li X. X. Zeng Y. Wang S. P. Sun Z. Y. Tang Y. Q. (2020). Dynamic change of bacterial community during dairy manure composting process revealed by high-throughput sequencing and advanced bioinformatics tools. Bioresour. Technol. 306:123091. doi: 10.1016/j.biortech.2020.123091, PMID: 32169511

[ref85] Zucconi F. Monaco A. Debertoldi M. (1981). Biological evaluation of compost maturity. Biocycle 22, 27–29.

