# Supplementary material for: Microbe-aided thermophilic composting accelerates manure fermentation
Source: Front Microbiol. 2024 Oct 25;15:1472922. doi: 10.3389/fmicb.2024.1472922 (PMC11544323; doi:10.3389/fmicb.2024.1472922)
Supplement: Supplementary file 1 [file Data_Sheet_1.docx]

**Supplementary information for**

**Microbe-aided thermophilic composting accelerates manure fermentation**

Likun Wang^13*^, Yan Li^1,2^, Xiaofang Li^1*^

^1^Center for Agricultural Resources Research, Institute of Genetics and Developmental Biology, Chinese Academy of Sciences, Shijiazhuang 050021, China

^2^ University of Chinese Academy of Sciences, Beijing 100049, China

^3^ Yancheng Institute of Soil Ecology, Yancheng 224051, China

*Correspondence to: xfli@sjziam.ac.cn (XL) and lkwang@sjziam.ac.cn (LW); No. 286 Huaizhong Rd, Shijiazhuang 050021, China


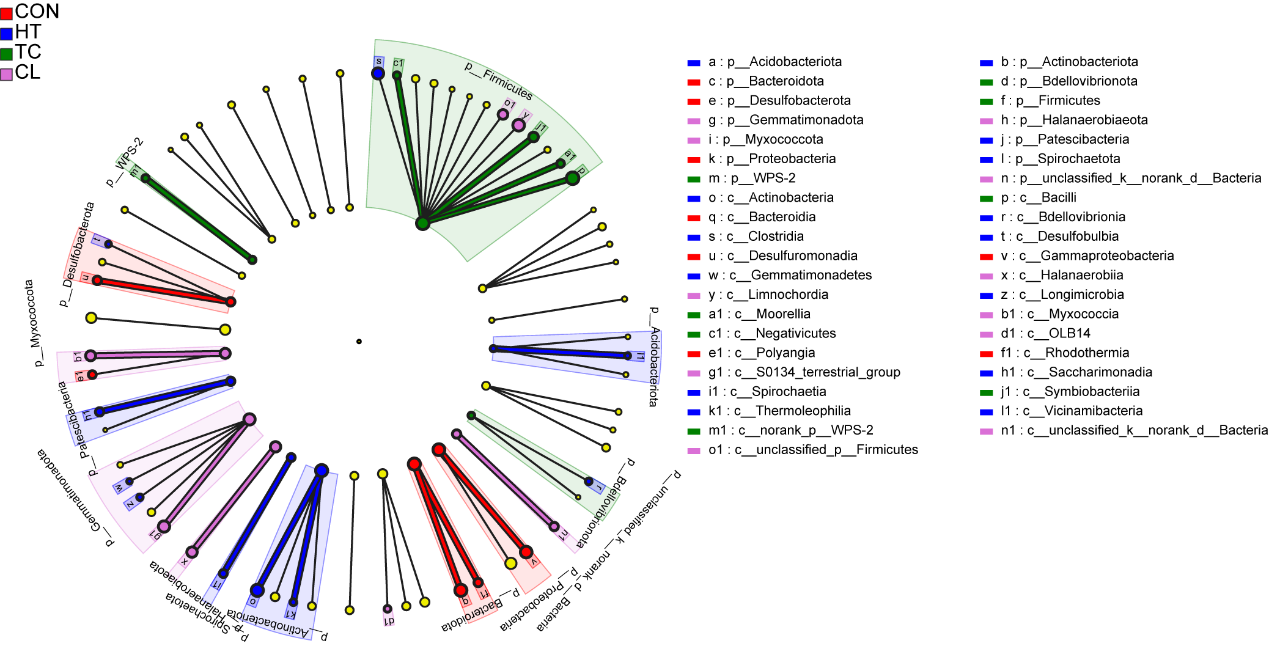


Suppl. Fig. 1 Lefse analysis of bacterial taxa among the four composting process from Phylum to Class level. Different colored nodes represent bacterial communities that are significantly enriched in their corresponding groups and have a significant impact on inter group differences; the light yellow nodes represent microbial communities that have no significant differences in different groups or have no significant impact on inter group differences.


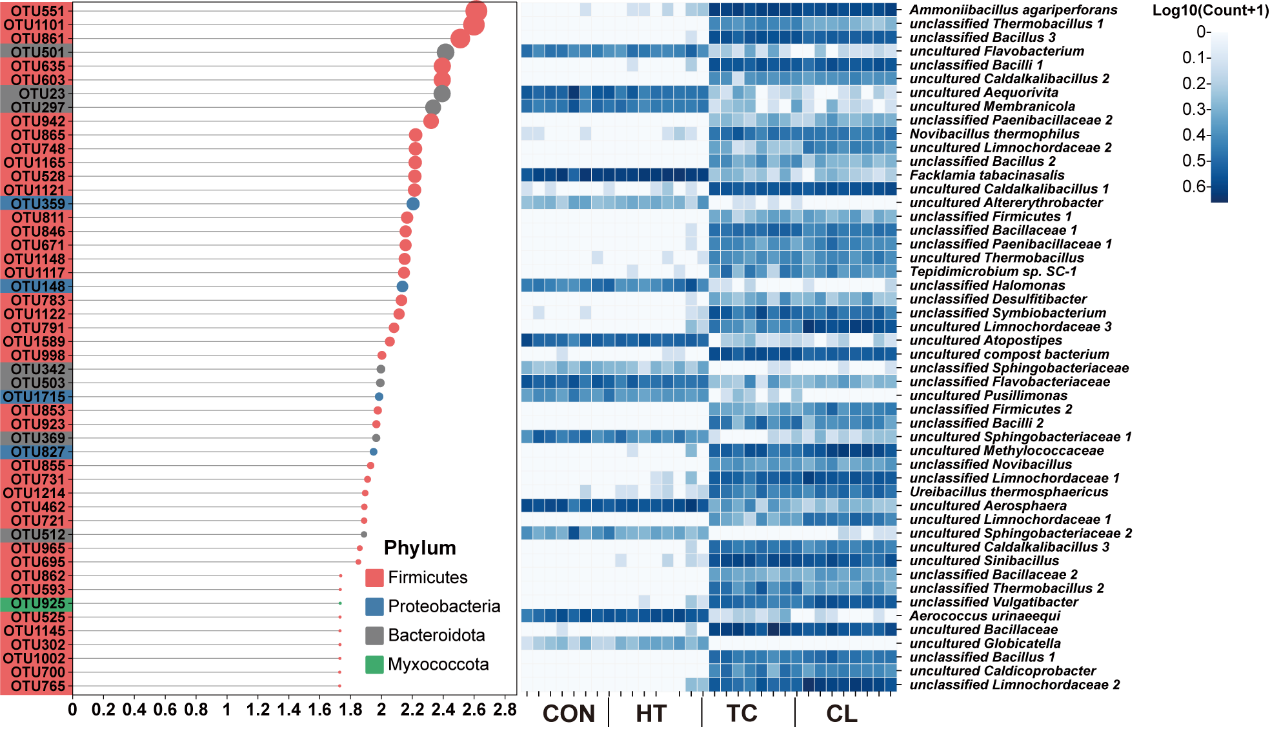


Suppl. Fig. 2 Random forest analysis of bacterial OTUs between the thermophilic and control groups. Lollipop graph shows the ranking of important OTUs and their impacts on the accuracy of random forest classification model. Heatmap shows the abundance of these important OTUS in each composting steps and their annotation.


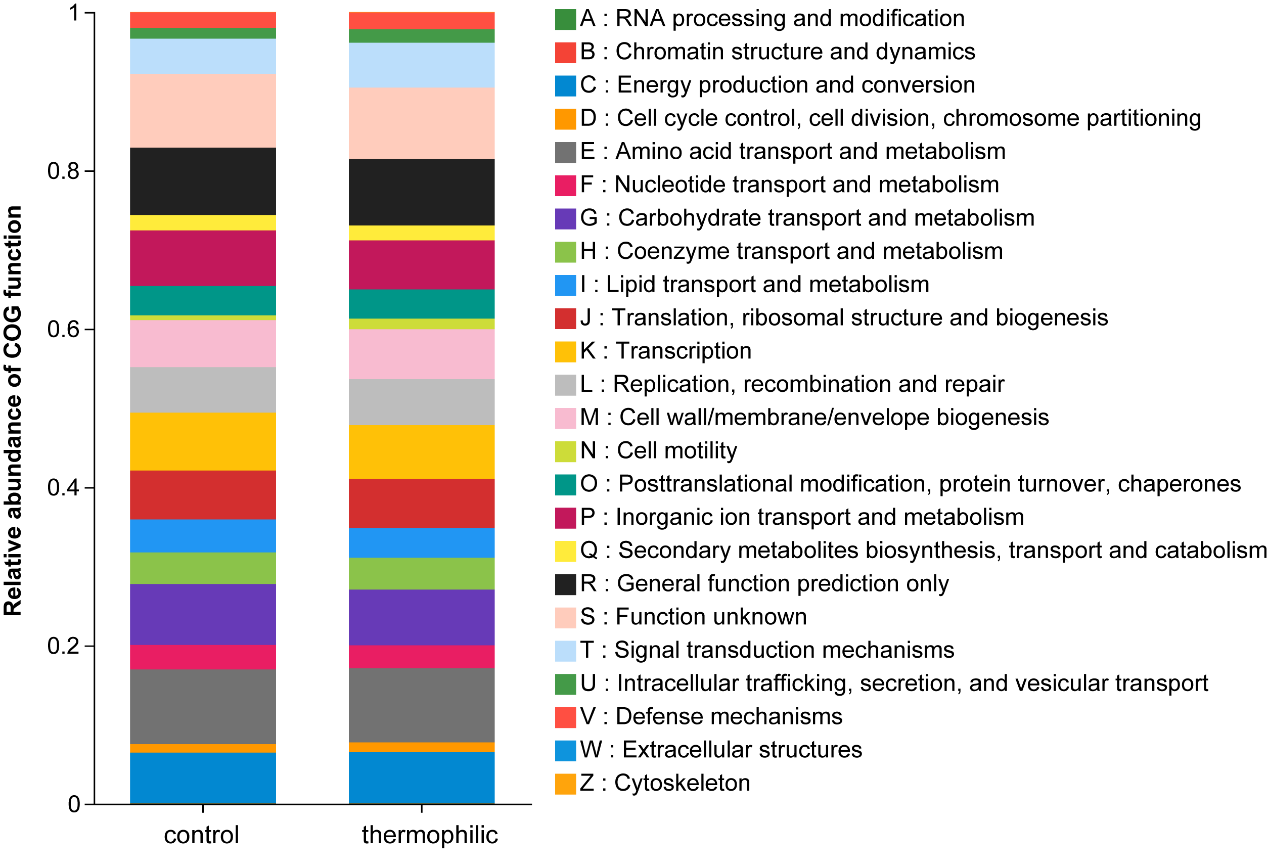


Suppl. Fig. 3 The abundance bacterial function in thermophilic and control group that analyzed through PICRUSt1. COG functional annotations are performed on the OTU to obtain annotation information at different levels of COG as well as abundance information of each function in different samples.


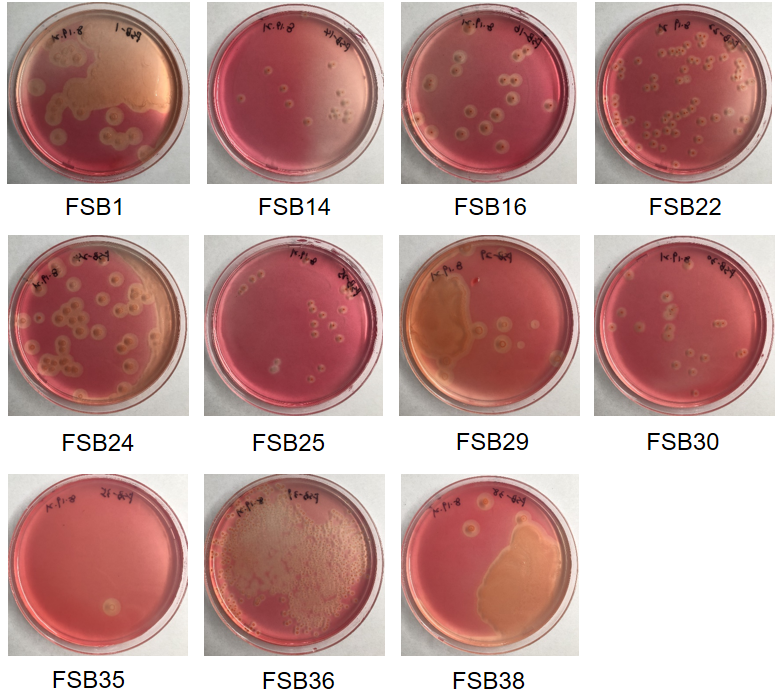


Suppl. Fig. 4 Congo red staining of eleven cellulase producing bacterial isolates. Bacterial isolates were incubated in solid medium for 18 h.


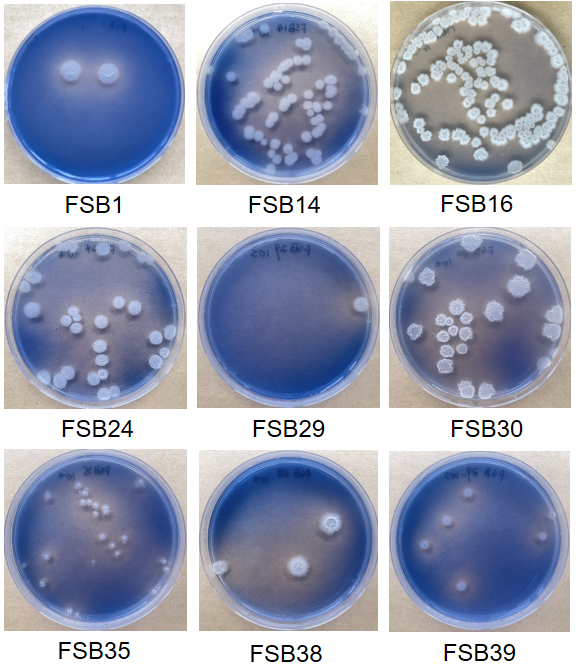


Suppl. Fig. 5 Aniline blue staining of nine ligninase producing bacterial isolates. Bacterial isolates were incubated in solid medium for 18 h.


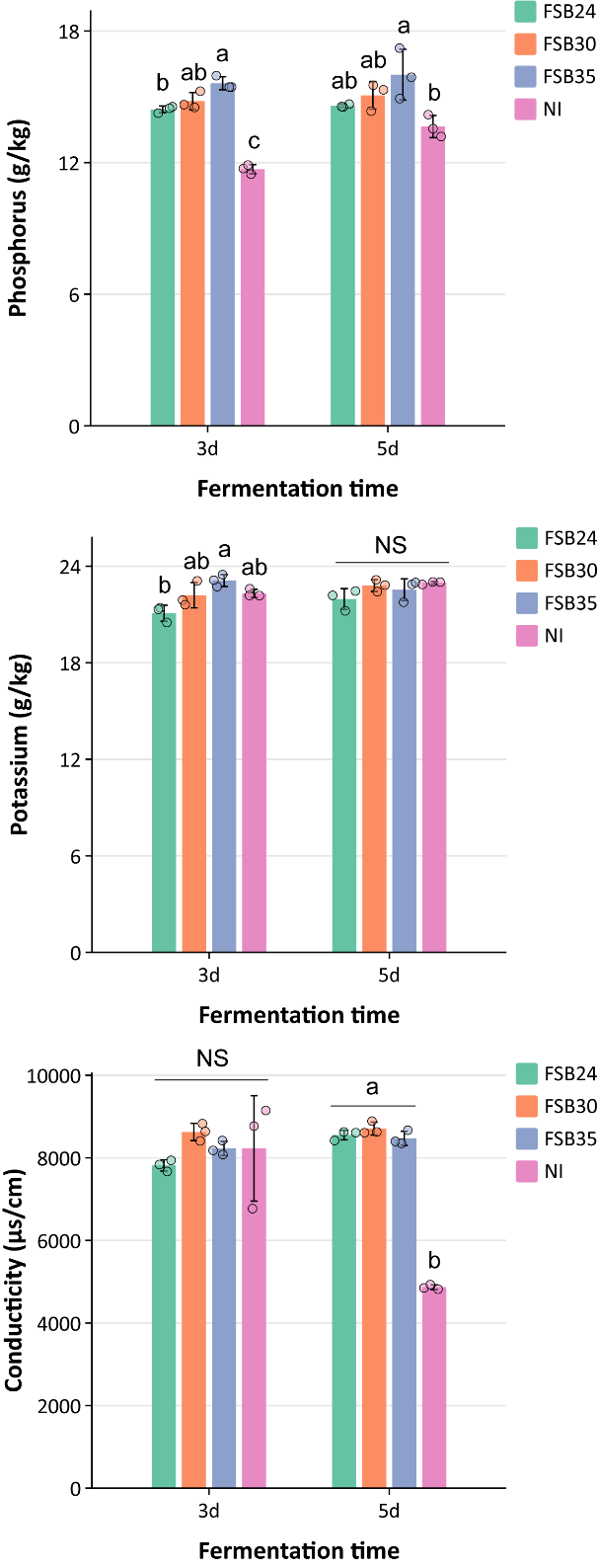


Suppl. Fig. 6 Influence of microbial inoculation on compost physiochemical characteristics after 3 days and 5 days of thermophilic stage. a) Total soluble phosphorus content in the compost inoculated with the three bacterial isolates, individually. b) Total potassium content in compost inoculated with the three bacterial isolates, individually. c) Conductivity index of the compost inoculated with the three bacterial isolates, individually. Bars indicate standard error of the mean. For a given duration of heat treatment, bars designated with the same letter indicate means that are not significantly different based on Fisher’s LSD analysis. NI: no inoculation.


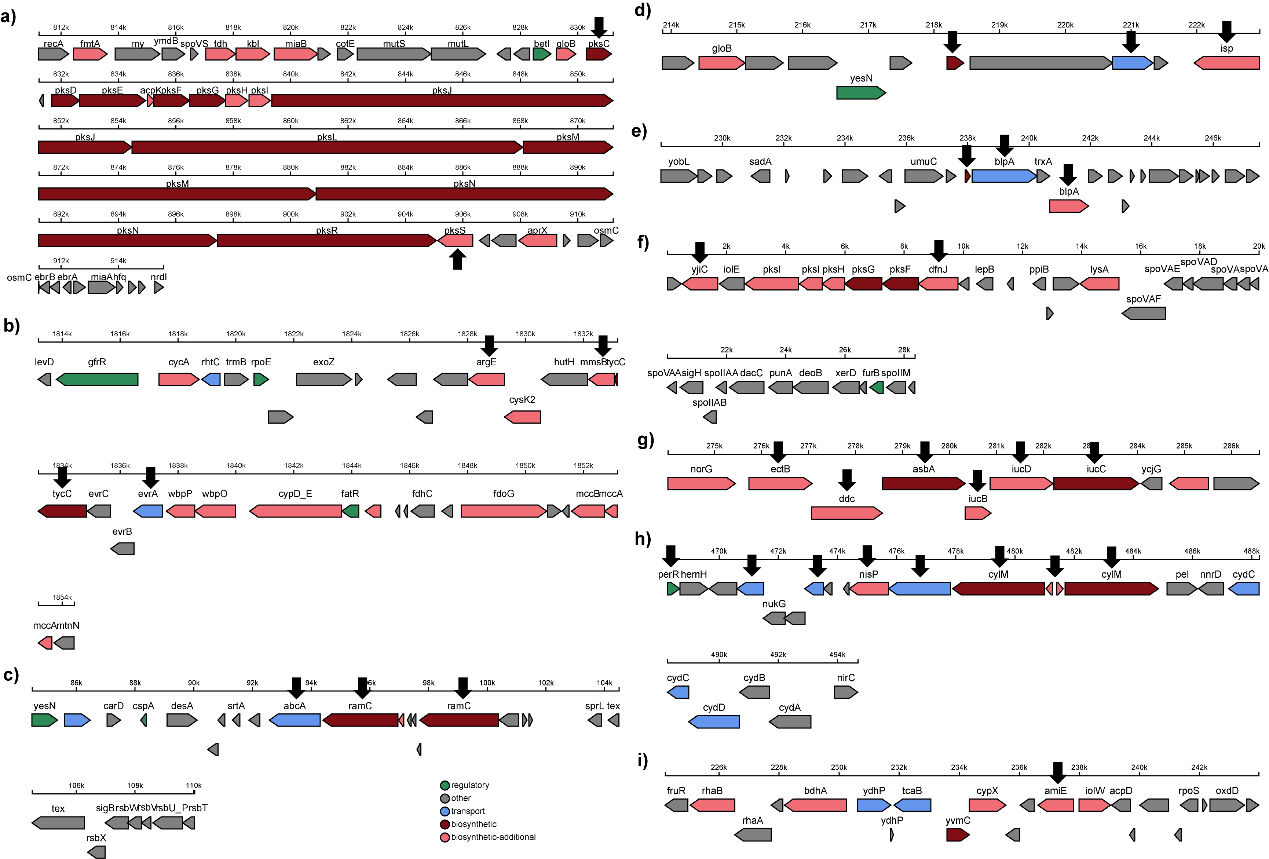


Suppl. Fig. 7 The unique genes found in the genomes of three bacterial isolates FSB24, FSB30, and FSB35, respectively, and the location of these genes in the gene cluster of secondary metabolite synthesis. a) to c) Gene clusters of secondary metabolite synthesis that contain unique genes in FSB24. d) to f) Gene clusters of secondary metabolite synthesis that contain unique genes in FSB30. g) to i) Gene clusters of secondary metabolite synthesis that contain unique genes in FSB35. Gene clusters were predicted through antismash. Black arrows pointed to the unique genes.

Table S1 The properties of raw livestock manure materials.

| Physicochemical properties | Average (n=3) |
| --- | --- |
| Organic matter | 543.82 g/kg |
| Total nitrogen | 32.09 g/kg |
| Total carbon | 332.46 g/kg |
| Total phosphorus | 15.68 g/kg |
| Total potassium | 26.07 g/kg |
| pH | 6.47 |
| Water content | 55% |
| Conductivity | 14660 μs/cm |

Table S2 Alpha diversity of bacterial communities in the four composting processes.

|  | Shannon | Simpsons | Sobs | Ace |
| --- | --- | --- | --- | --- |
| Control | 4.42±0.36 | 0.04±0.02 | 746.63±189.51 | 1148.04±284.30 |
| HT | 4.65±0.17 | 0.03±0.01 | 938.25±91.49 | 1289.46±66.97 |
| TC | 4.27±0.24 | 0.04±0.01 | 647.88±95.22 | 920.29±75.96 |
| CL | 4.31±0.10 | 0.03±0.00 | 623.00±37.15 | 865.21±112.47 |
